# Safety and neuroprotective efficacy of the VCP inhibitor ML240 in large-animal and human retinal explants: a preclinical ex vivo study

**DOI:** 10.1186/s12916-025-04610-0

**Published:** 2026-01-10

**Authors:** Ana-Cristina Almansa-García, Angela Armento, Bowen Cao, Anne-Sophie Petremann-Dumé, Stefano Salmaso, Paolo Caliceti, Cristine Henes, Sylvie Bolz, Ellen Kilger, Daniela Süsskind, Marius Ueffing, Blanca Arango-Gonzalez

**Affiliations:** 1 Institute for Ophthalmic Research, Centre for Ophthalmology, University of Tübingen, Tübingen, Germany; 2 Graduate Training Centre of Neuroscience, University of Tübingen, Tübingen, Germany; 3 Department of Pharmaceutical and Pharmacological Sciences, University of Padova, Padua, Italy; 4 Department of Ophthalmology, University of Tübingen, Tübingen, Germany

**Keywords:** VCP/p97, ML240, Retina, Retinal explants, Retinal degeneration, Neuroprotection, Drug delivery, Primate retina, Human retina, Macula

## Abstract

**Background:**

Retinal degenerative diseases represent a complex global health problem due to their significant impact on patients’ daily lives and their highly heterogeneous pathogenesis, which challenges therapeutic development. Despite this complexity, many diseases, such as retinitis pigmentosa (RP) and age-related macular degeneration (AMD), share common features, including disrupted proteostasis, oxidative stress, and inflammatory responses, eventually leading to photoreceptor (PR) degeneration and vision loss. The inhibition of valosin-containing protein (VCP) has emerged as a promising mutation-independent therapeutic strategy for RP. However, clinical translation requires rigorous validation in models that closely reflect human retinal physiology.

**Methods:**

Organotypic retinal explants from porcine, macaque, and human donors were placed in an in vitro culture setup and treated with ML240, a selective VCP inhibitor, delivered either as a free compound or encapsulated in mPEG_5kDa_-cholane. Photoreceptor survival was assessed via TUNEL assay, outer nuclear layer (ONL) row quantification, and immunostaining. Retinal inflammation was evaluated by microglial staining. A dose–response study was performed to determine safety margins across species, and additional retinal markers were used to assess the preservation of non-photoreceptor retinal cell populations.

**Results:**

Porcine retinal explants exhibited progressive photoreceptor degeneration under ex vivo conditions. Treatment with ML240, particularly when formulated with mPEG_5kDa_-cholane, significantly reduced photoreceptor cell death and microglial activation. Macaque and human explants exhibited minimal to no signs of degeneration. Treatment did not affect morphological or histological features of the explant, demonstrating the safety of ML240 in the primate retina.

**Conclusions:**

VCP inhibition via ML240 demonstrates an uncompromised safety profile in porcine, macaque, and human retinal explants. In addition, the neuroprotective activity of ML240 was evident in porcine tissue. Formulation with mPEG_5kDa_-cholane enhances the overall performance of the compound, supporting its use for future clinical application as a mutation-independent therapeutic approach for retinal degenerative diseases.

**Graphical Abstract:**

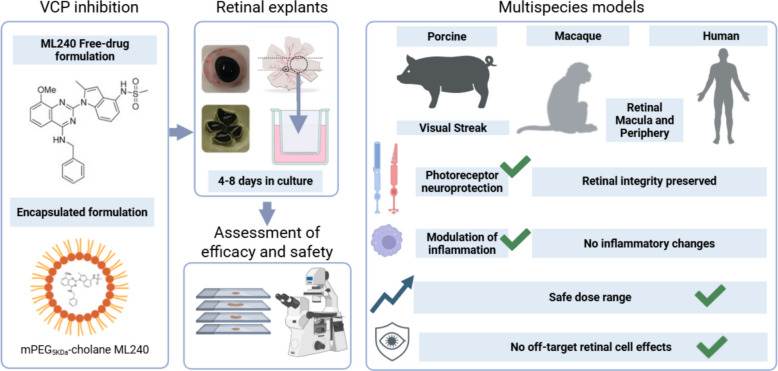

**Supplementary Information:**

The online version contains supplementary material available at 10.1186/s12916-025-04610-0.

## Background

Vision plays a fundamental role in daily life. From reading to driving, we depend greatly on this sense; consequently, impairments in visual function have a profound impact on quality of life. Visual information is received and processed by the retina, a specialized part of the central nervous system (CNS), composed of neuronal layers that convert light into neural signals, which are then transmitted to the brain [1, 2].

The retina is affected by a wide range of diseases with high variability in etiology, clinical manifestations, and regional distribution [3, 4]. This heterogeneity represents a major challenge for therapy development and translation. Notably, fewer than 10% of clinical trials succeed globally [5, 6], underscoring the critical importance of a robust preclinical phase to prevent late-stage failure and associated cost escalation [6, 7]. Within this context, the selection of appropriate animal models is a critical component of the translational pipeline.

Although retinal structure is generally conserved across mammals, important species-specific differences exist that reflect evolutionary and behavioral adaptations [1, 8]. Murine models are most commonly used in ophthalmic research due to their genetic manipulability, availability of relevant disease mutations [9], and low cost. However, mouse retinas differ considerably from human retinas in cellular composition and spatial organization. Mice, being nocturnal, possess a rod-dominated retina without a fovea or foveal avascular zone (FAZ) [8, 10, 11]. In contrast, larger animals, such as pigs (*Sus scrofa*) [9, 12, 13], dogs (*Canis lupus familiaris*) [14], and non-human primates (NHPs) [15], have retinas more similar to those of humans. These species exhibit higher cone densities and, in the case of NHPs, a fovea, making them suitable for modeling diseases affecting the macula, such as age-related macular degeneration (AMD) and Stargardt disease [16]. Nonetheless, the use of large animals introduces technical, ethical, and economic challenges [15].

Translational failures in retinal therapies often have two main causes: insufficient efficacy or toxicity not detected in traditional animal models [17, 18]. As such, the use of complementary large-animal and human-derived models can mitigate this risk [17]. Organotypical retinal explants from pigs [19], NHPs [20], or human donors [21–23] offer a highly suitable intermediate platform that enables detailed safety and efficacy assessments while preserving the complex architecture of the retina.

We previously showed that pharmacological inhibition of valosin-containing protein (VCP) can attenuate the progression of certain forms of inherited retinal diseases (IRDs) [24–26]. VCP is a hexameric ATPase involved in proteostasis, autophagy, and apoptosis regulation [27, 28], and has been implicated in neurodegenerative conditions, such as frontotemporal dementia and Huntington’s disease [27]. In the diseased retina, VCP activity contributes to ER stress and perturbs proper proteostasis [24], which is a shared hallmark of IRD-associated degeneration.

Preclinical studies in *Drosophila melanogaster* [29] and rodent models (*Rattus norvegicus*, *Mus musculus*) [24, 25] with rhodopsin mutations (e.g., P23H) have shown that inhibition of VCP via small molecules such as ML240 [30] preserves photoreceptor structure and function. However, the translatability of these findings to human physiology and their safety across retinal cell types remains uncertain.

The present study addressed this gap by evaluating the safety and neuroprotective potential of ML240 using organotypic explants from pigs (*Sus scrofa*), macaque (*Macaca mulatta)*, and human (*Homo sapiens*) retina. ML240 was tested both as a free compound and encapsulated in methoxy polyethylene glycol 5 kDa-cholane (mPEG_5kDa_-cholane) nanoparticles [31], a delivery strategy designed to avoid dimethyl sulfoxide (DMSO)-associated toxicity and increase drug efficacy. Photoreceptor viability, microglial reactivity, dose tolerance, and preservation of inner retinal neurons were analyzed across species. The results of this study confirm a comprehensive preclinical safety profile of ML240, supporting its further development as a mutation-independent therapeutic candidate for retinal degenerative diseases.

## Methods

### Animal and human material

Porcine eyes were obtained from a local slaughterhouse and immediately transported on ice to the laboratory. Retinal explants were prepared from 6-month-old pigs (average weight ~ 100 kg) within 2–3 h post-enucleation, following a previously described protocol [32].

Eyes from an adult macaque monkey, which had not been subjected to any systemic treatment or procedure, were provided postmortem by the Cognitive Neurology Laboratory at the Hertie Institute for Clinical Brain Research (Tübingen) after a terminal experiment approved by the Institutional Animal Welfare Office of the University of Tübingen (approval number: N7/18) and conducted in accordance with the German Animal Protection Law (Tierschutzgesetz). Eyes were transported in cold phosphate-buffered saline (PBS) and dissected within 20–30 min after enucleation.

All procedures involving porcine and macaque tissues were performed in accordance with institutional guidelines and were approved by the local competent authority (Landratsamt Tübingen), under the registration number DE 08 416 1157 21, authorizing the use of category 1 animal by-products for research purposes (Regulation (EC) No. 1069/2009; Regulation (EU) No. 142/2011).

Human eyes were obtained with informed consent from patients diagnosed with uveal melanoma who agreed to donate their eyes after enucleation surgery at the University Hospital of Tübingen. Tissues were transported in cold PBS and dissected for retinal culture within 15–30 min post-enucleation. Human tissue use was approved by the local ethics committee (reference number: 185/2021BO2).

### Organotypic culture

Eyecups from pigs, macaques, and humans were briefly sterilized by immersion in 70% ethanol for 10 s, followed by PBS rinsing under aseptic conditions. Surrounding muscle tissue was removed using Westcott scissors. The anterior segment was excised by making a scleral incision below the ciliary body with a scalpel (Feather Safety Razor Co., LTD, 02.001.30.023), then completed with scissors. The vitreous body was carefully removed to preserve retinal integrity.

Retinal explants were obtained using sterile biopsy punches (5 mm for pig eyes, 4 mm for macaque and human eyes; PFM Medical, 48,501 and 48,401). In pigs, the visual streak (avascular, cone-rich region) was identified, and two explants per eye were collected to allow for paired treatment–control comparisons. In macaque and human eyes, explants were obtained from the retina after removal of the anterior segment and vitreous, with identification of the macular region in humans.

Explants were placed photoreceptor side down onto 0.4 µm pore-size polycarbonate membranes (Corning Life Sciences, CLS3412). Cultures were incubated in serum-free complete culture medium consisting of Neurobasal™-A (ThermoFisher, 10,888,022), 2% B-27 supplement (ThermoFisher, 17,502–044), 1% N2 supplement (ThermoFisher, 17,502–048), 0.8% Glutamax (Gibco, 35,050–061), and 1% Antibiotic–Antimycotic Solution (Gibco, 15,240,062). Incubation was performed at 37 °C with 5% CO_2_ for up to 8 days in vitro (DIV), with medium changed every other day.

### Treatments

ML240 (Tocris, Bio-Techne GmbH, 5153) was dissolved in dimethyl sulfoxide (DMSO, Roth, A994.1) to obtain a 5 mM stock solution. This was further diluted in pH 5-adjusted Neurobasal A medium to prepare a 1 mM ML240 working solution containing 20% DMSO. A 20 µl aliquot of this solution was added to 1 ml of culture medium (final concentration: 20 µM ML240 or 0.4% DMSO). Medium and treatments were refreshed every other day.

The mPEG-cholane nanoparticles were synthesized according to previously published protocols [31], and ML240-loaded nanoparticles were generated based on the “film hydration” method as previously described [32]. Encapsulated ML240 (5 µM) or empty nanoparticles (0.25 mg/ml) were administered directly on top of the GCL (Ganglion cell layer) side of the retinal surface at culture onset (Table 1).

**Table 1:** Treatment conditions

Condition	Vehicle	Application method	Final concentration	Treatment frequency
ML240 (free)	0.4% DMSO	20 µL of 1 mM ML240 in 1 mL medium	20 µM ML240	Every second day
Vehicle control (DMSO)	0.4% DMSO	20 µL of 20% DMSO in 1 mL medium	0.4% DMSO	Every second day
ML240 (encapsulated)	0.25 mg/mL mPEG₅_kDa_-cholane nanoparticles	Single drop on retina	5 µM ML240	Single application
Vehicle control (nanoparticles)	0.25 mg/mL mPEG₅_kDa_-cholane nanoparticles	Single drop on retina	0.25 mg/mL	Single application

### Histology

Retinal explants were fixed in a 4% paraformaldehyde solution in 0.1 M phosphate buffer (PB, pH 7.4) for 45 min and cryoprotected in a sucrose gradient (10%, 20%, and 30%). Tissues were embedded in cryomatrix (Tissue-Tek® O.C.T. Compound, Sakura® Finetek, VWR, 4583) and snap-frozen in liquid nitrogen. Radial Sects. (14 µm) were cut, air-dried at 37 °C, and stored at − 20 °C.

### TUNEL assay

Cell death was assessed using an in situ cell death detection kit (Roche, 11,684,795,910) conjugated with fluorescein isothiocyanate (FITC), according to the manufacturer’s protocol. Nuclei were counterstained with DAPI (1:5000 in PBS; Sigma, D-9542) for 5 min. Slides were mounted using Fluoromount-G (Electron Microscopy Sciences, 17,984–25).

### Immunohistochemistry

Sections were permeabilized and blocked with 10% normal goat serum (PAA-Labs,311–053) or 10% donkey serum (Sigma, D9663), plus 1% bovine serum albumin (PAA-Labs, K41-001) in 0.1% PBST (PBS + 0.1% Tween) for 1 h at room temperature. Primary antibodies were incubated overnight at 4 °C (Rabbit anti-iba1 (polyclonal), 1:500, Fujifilm Wako Chemicals, 019–19741; mouse anti-rhodopsin (monoclonal), 1:350, Sigma-Aldrich, MAB5316; rabbit anti-M opsin (polyclonal), 1:200, Sigma-Aldrich, AB5405; mouse anti-cone arrestin (monoclonal), 1:500, Millipore, MABN2636; mouse anti-PKCα (monoclonal), 1:300, Novus biologicals, NB600-201SS; rabbit anti-RNA-binding protein with multiple splicing (RBPMS) (polyclonal), 1:100, Novus biologicals, NBP2-20,112; mouse anti-calretinin (monoclonal), 1:300, Chemicon, MAB1568; chicken anti-calbindin (polyclonal), 1:500, Novus Biologicals, NBP2-50028SS).

Secondary antibodies (Alexa Fluor™568 dye-conjugated goat anti-mouse IgG, 1:500, ThermoFisher, A11031; Alexa Fluor™ 568 dye-conjugated goat anti-rabbit IgG, 1:500, Molecular Probes, A11036; or Alexa Fluor™ 488 dye-conjugated goat anti-chicken, 1:500, Invitrogen, A11039) were applied for 1 h at room temperature, followed by DAPI counterstaining and mounting.

### Microscopy and image analysis

Z-stacks were acquired using a Zeiss Axio Imager Z1 ApoTome microscope (20 × objective) and analyzed with Zen Blue 3.7. Samples from the same set of experiments were always stained and imaged on the same day, with identical microscope settings to avoid technical variability. Three to four sections per sample were imaged. ONL rows were counted manually based on DAPI labeling. TUNEL-positive cells were quantified as a percentage of total ONL cells and normalized to the corresponding controls. The total ONL cell number was calculated by dividing the ONL area by the average photoreceptor nucleus size, which was obtained by measuring the area of at least six photoreceptor nuclei from different regions of the retinal sections using the contouring tool in Zen Blue 3.7. The mean value of these measurements was used as the average nuclear size. Iba1 immunofluorescence intensity was quantified using the contouring tool in Zen Blue 3.7 as a relative measure of microglial activation. Mean fluorescence intensity was measured either across the full retinal section or restricted to the ONL, and values were normalized to the corresponding controls. Cone density was determined by manually counting the number of cone photoreceptors within three independent 100-µm regions per image, and expressed as the number of cones per 100 µm of retinal section. To illustrate and facilitate retinal layer identification, DAPI staining was added to the left part of the representative immunostaining images.

#### Western blot

Retinal explants were collected in lysing kit tubes (P000933-LYSK0-A, Precellys) with 100 µl of lysing buffer (87,787, Thermo Fisher Scientific) to which we added protease and phosphatase inhibitor (1,861,281, Thermo Fischer Scientific), and lysates were homogenised using a Precellys 24 tissue homogenizer. After 20 min of centrifugation at 4 °C, supernatants were collected and protein concentration was quantified by Bradford (500–0006, Biorad). 20 µg of proteins were mixed with 1 × Laemmli sample buffer and separated by electrophoresis in 8–16% SDS-PAGE gels (XP08165Box; Thermo Fisher Scientific) and transferred to PVFD membranes (0,45 µm, 10,600,023, Cytiva). Membranes were blocked in 5% milk in TBST (Tris-buffered saline with 1% Tween 20) and incubated with the corresponding primary antibodies (anti-Iba1 1:1000, 019–19741, FUJIFILM Wako; anti-β-actin 1:2000, 3700S, Cell Signalling) overnight at 4 °C. Afterwards, membranes were incubated for 1 h at room temperature with horseradish peroxidase-coupled (HRP) secondary antibodies (anti-mouse 1:2000, 7076P2, Cell Signalling; anti-rabbit 1:2000, 7074P2, Cell Signalling). ECL Plus chemiluminescent and chemifluorescent HRP detection reagent (32,132, Thermo Fisher Scientific) was then applied and detected using a FusionFX instrument (Vilber Lourmat, France). Band intensity was quantified using ImageJ software, and relative protein expression levels were determined based on the control group.

### Statistics

Analyses were performed using GraphPad Prism version 10. Data were presented as box plots to illustrate distribution and variability between experimental groups. Filled circles in the plots represent biological replicates. For the porcine experiments, each data point shown as a filled circle in the figures represents an independent retina from a different animal (*n*), with at least six biological replicates per condition (*n* = 6–8).

In macaque and human experiments, where tissue availability was limited, multiple retinal explants were obtained from each donor retina. These explants were analyzed as individual samples and are represented as filled circles in the figures (macaque, *n* = 3; human, *n* = 4–5).

Explants from the macaque retina were prepared from both eyes of a single adult donor. For human experiments, explants treated with free ML240 originated from two different donors, whereas those treated with encapsulated ML240 were derived from one retina of a single donor. For each explant, multiple radial sections were collected, and several non-overlapping regions were imaged per section and analyzed. These individual measurements are shown as open circles in the plots and represent technical replicates used to calculate means for each biological replicate.

Group comparisons were performed using paired or unpaired *t*-tests, depending on the experimental design. For experiments involving more than two groups, one-way analysis of variance (ANOVA) was used, followed by Tukey’s multiple comparisons test. A *p*-value less than 0.05 was considered statistically significant.

## Results

### VCP inhibition by ML240 is well tolerated and promotes photoreceptor protection in wild-type porcine retinal explants

**Fig. 1:**
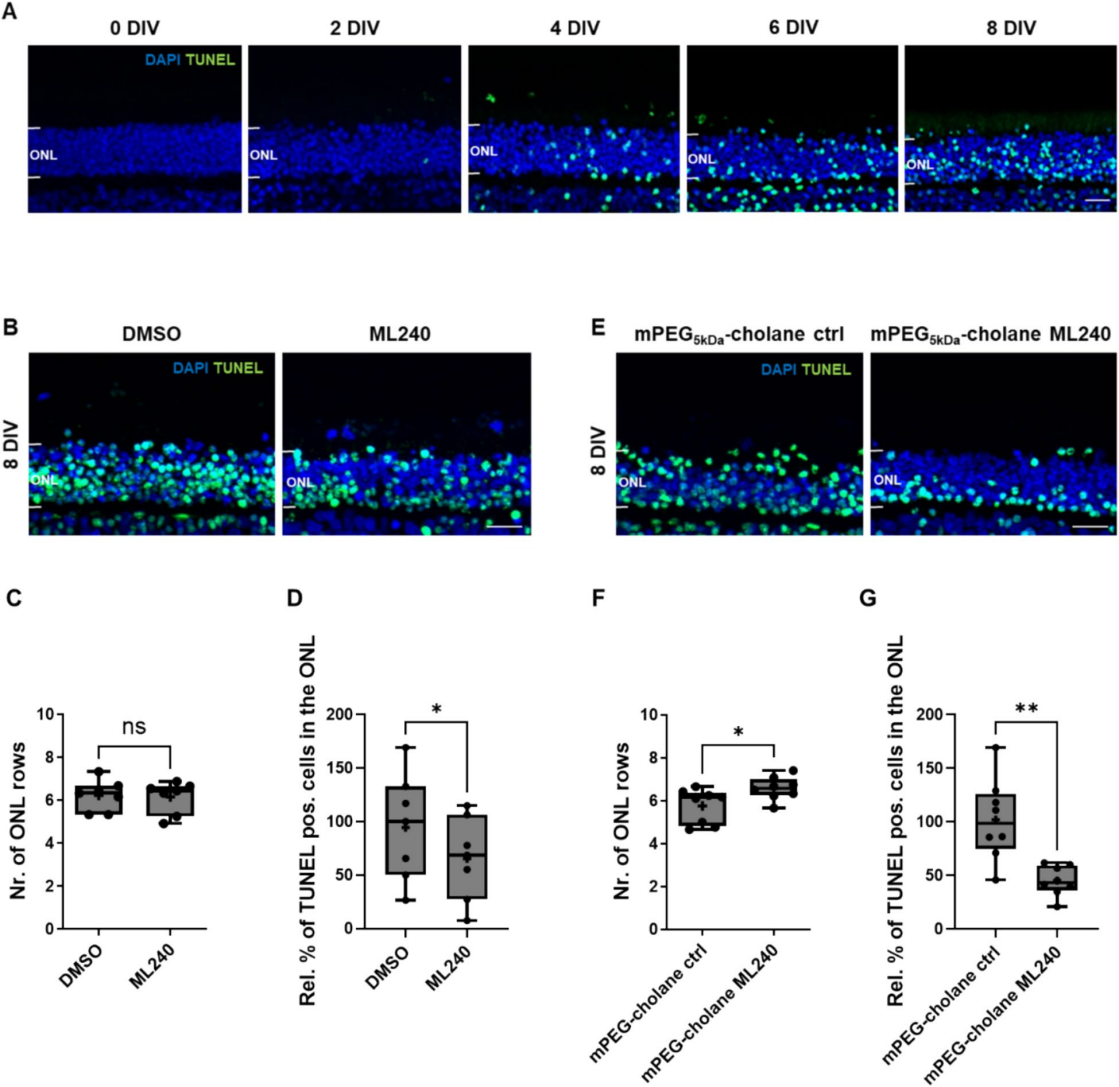
VCP inhibition improves photoreceptor survival in porcine retinal explants in vitro*.* **A** Progressive photoreceptor degeneration in porcine explants over time. TUNEL assay was used to detect photoreceptors undergoing cell death, with DAPI counterstaining for nuclear visualization. **B** Representative images of explants treated for 8 days (8 DIV) either with ML240 (20 µM) or DMSO control, and **E** with mPEG_5kDa_-cholane-encapsulated ML240 (5 µM) or nanoparticle vehicle control (0.25 mg/mL). Neuroprotective effects were assessed by quantification of photoreceptor cell rows in the ONL (**C**, **F**) and the percentage of TUNEL-positive cells in the ONL (**D**, **G**). Statistical differences between groups were evaluated using paired *t*-tests (*n* = 7–8; * *p* < 0.05; ** *p* < 0.01). Scale bar: 20 µm

To analyze the effect of ML240 on both rods and cones, the cone-rich avascular visual streak region of the porcine retina was examined. Its structural and physiological features resemble the parafovea of the human retina. Untreated porcine retinal explants showed progressive degeneration in vitro, as indicated by a time-dependent increase in the percentage of TUNEL-positive cells in the outer nuclear layer (ONL), particularly after 6 and 8 days in vitro (DIV) (Fig. 1A), reflecting ongoing photoreceptor cell death. As expected for this ex vivo model, porcine retinal explants displayed degeneration affecting multiple retinal layers, not only the photoreceptor layer (Additional file 1: Fig. S1). Nevertheless, our quantitative analyses focused on the outer nuclear layer (ONL), given the previously established role of VCP inhibition in photoreceptor protection in murine models of RP.

When treated with ML240 either in its free form (20 µM, dissolved in DMSO) or encapsulated in mPEG_5kDa_-cholane nanoparticles (5 µM), a formulation designed to mitigate DMSO-associated toxicity [33], porcine retinal explants showed no signs of cytotoxicity or structural disorganization. Treatment with either form significantly decreased the percentage of TUNEL-positive cells in the ONL (Fig. 1D, G), indicating improved photoreceptor survival.

Notably, only encapsulated ML240 resulted in an increased number of ONL cell rows at this time point (Fig. 1E), further supporting its neuroprotective activity. No changes were observed in the rhodopsin distribution between the ONL and the outer segments (OS) (Additional file 1: Fig. S2), nor were differences detected in cone photoreceptor survival (Additional file 1: Fig. S3).

### VCP inhibition by mPEG_5kDa_-cholane-encapsulated ML240 effectively reduces microglial activation in porcine retinal explants

**Fig. 2:**
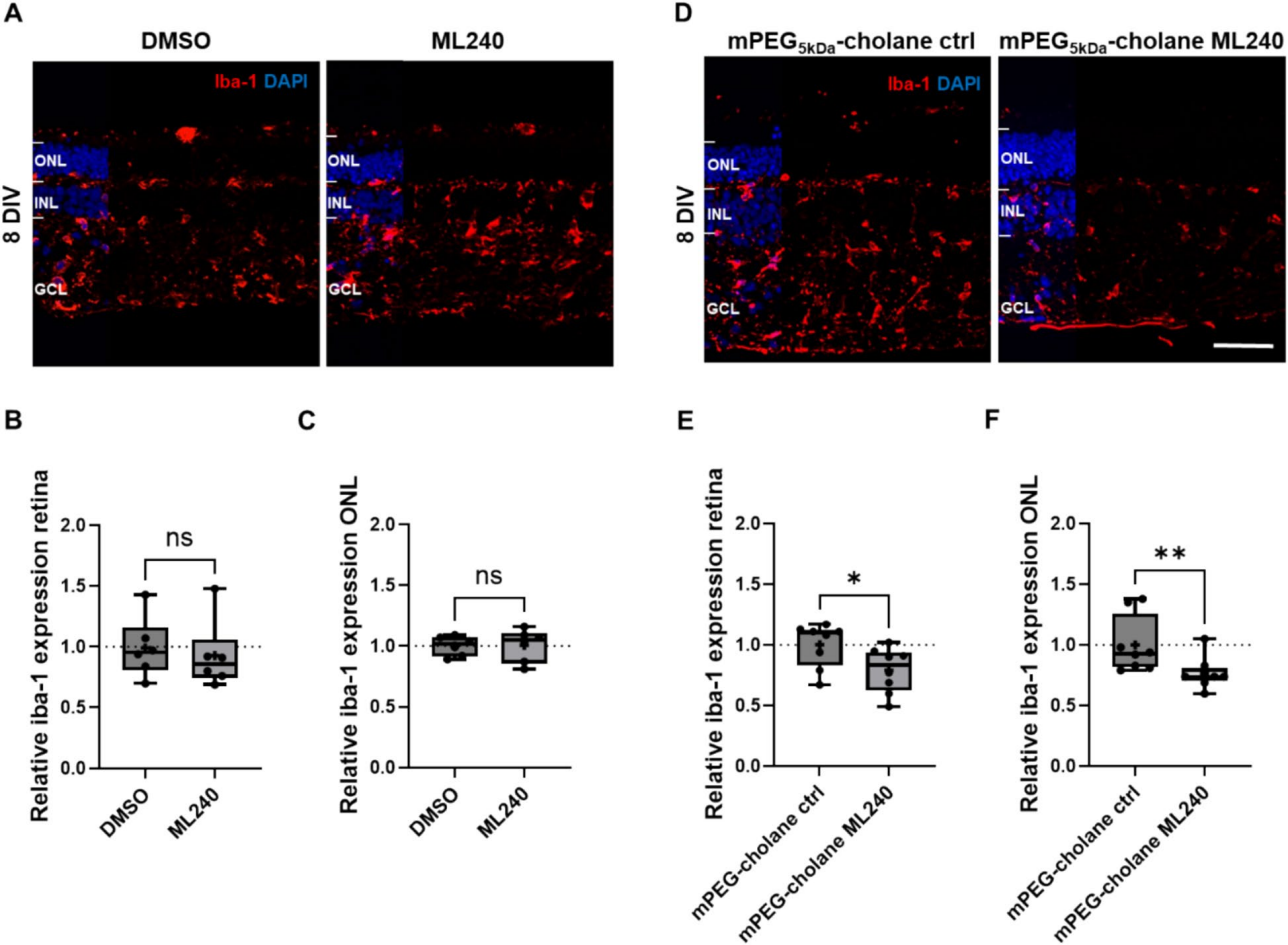
mPEG_5kDa_-cholane-encapsulated ML240 reduces microglial Iba1 expression in porcine retinal explants. Retinal explants were cultured for 8 DIV and treated with either ML240 (20 µM) or DMSO control, and mPEG_5kDa_-cholane-encapsulated ML240 (5 µM) or mPEG_5kDa_-cholane vehicle control (0.25 mg/mL). Retina sections were immunostained for Iba1 to assess microglial activation and DAPI for nuclei counterstaining. Representative images show Iba1 expression in explants treated with free ML240 or DMSO (**A**), and mPEG_5kDa_-cholane ML240 or nanoparticle vehicle control (**D**). Fluorescence intensity of Iba1 was quantified across the entire retinal section (**B**, **E**) and specifically within the ONL (**C**, **F**), normalized to the respective controls. Statistical differences were evaluated using paired *t*-tests (*n* = 6–8; * *p* < 0.05). Scale bar: 50 µm

Retinal and photoreceptor degeneration are known to activate resident microglia, triggering a macrophage-like inflammatory response that may exacerbate disease progression [34, 35]. This inflammatory cascade is thought to contribute to the progression of retinal pathology [34]. Here, microglial reactivity was assessed by Iba1 immunostaining in porcine explants cultured for 8 DIV under different treatment conditions (Fig. 2A, D). No significant differences in Iba1 expression were observed between explants treated with DMSO and those treated with free ML240 (20 µM) (Fig. 2B, C), suggesting that the free compound does not trigger an inflammatory response. In contrast, explants treated with mPEG_5kDa_-cholane-encapsulated ML240 (5 µM) displayed a significant reduction in Iba1 expression, both across the entire retinal section (Fig. 2E) and specifically within the ONL area (Fig. 2F). Iba1 protein expression decreased in mPEG5kDa-cholane-encapsulated ML240-treated explants, which was further confirmed by western blot (Additional file1: Fig. S4; Additional file 2). These observations reinforce the immune safety profile of ML240 and suggest that its encapsulated formulation may attenuate microglial activation, possibly due to improved delivery and reduced toxicity of the vehicle. Collectively, the results indicate that ML240, particularly in its encapsulated form, not only avoids triggering inflammation but may actively mitigate inflammatory processes associated with retinal stress.

### VCP inhibition by mPEG_5kDa_-cholane-encapsulated ML240 displays a wide safety margin in porcine retinal explants

**Fig. 3:**
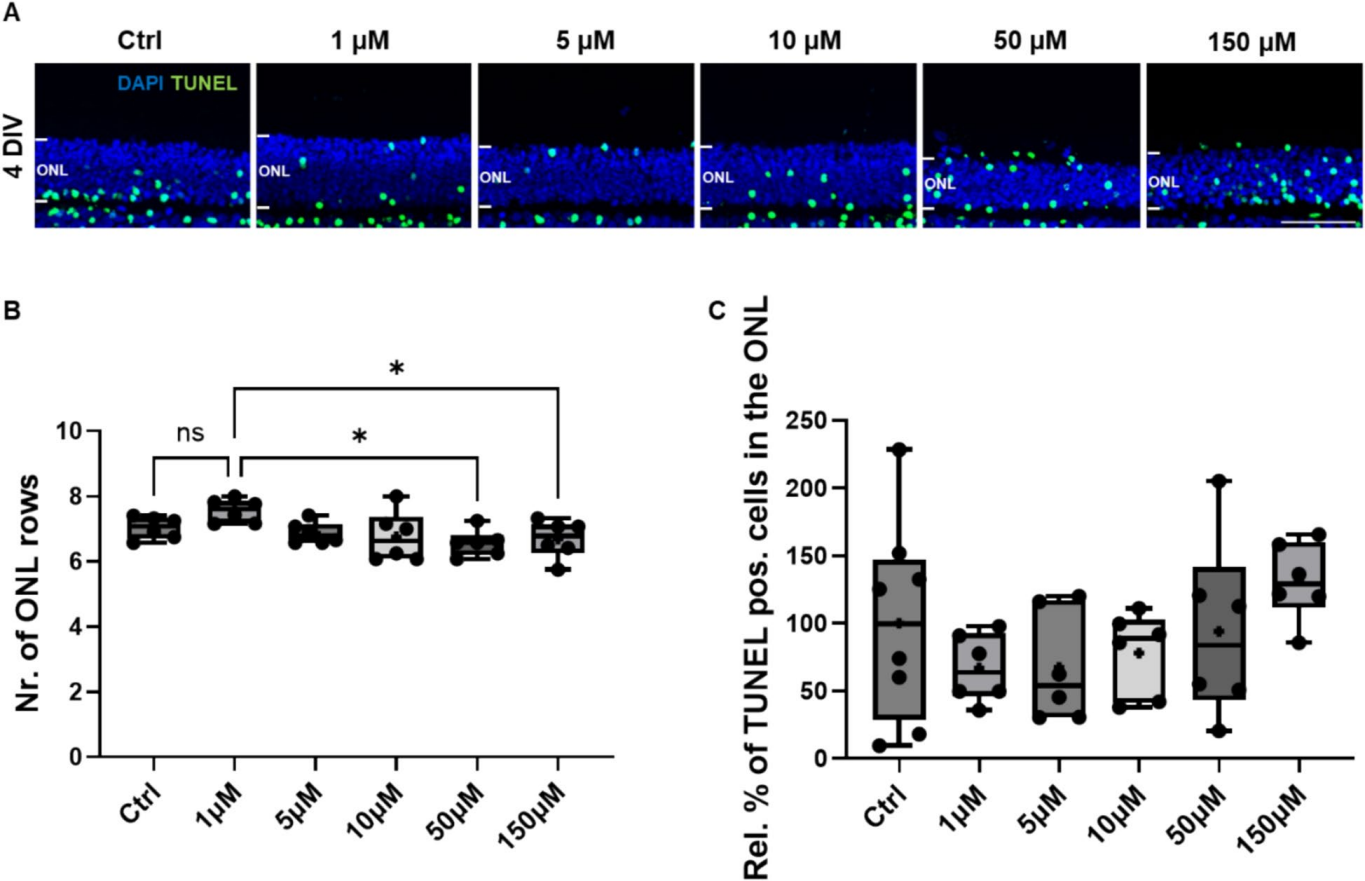
Dose-dependent effects of mPEG_5kDa_-cholane-encapsulated ML240 on photoreceptor survival in porcine retinal explants. **A** Retinal explants were cultured for 4 DIV and treated with increasing concentrations of mPEG_5kDa_-cholane-encapsulated ML240 (1 µM, 5 µM, 10 µM, 50 µM, 150 µM). Empty mPEG_5kDa_-cholane nanoparticles were used as controls at the same concentration as the highest dose used (7.5 mg/mL). TUNEL assay was used to visualize photoreceptors undergoing cell death, and DAPI was used to counterstain the retinal nuclei. Retinal evaluation was performed by quantification of ONL nuclei (**B**) and percentage of TUNEL-positive cells in the ONL (**C**). Statistical analysis was performed using one-way ANOVA followed by Tukey’s multiple comparisons (*n* = 6; * *p* < 0.05). Scale bar: 50 µm

To further investigate the tolerability of ML240 and define its safety window, a dose-escalation study was performed with the mPEG_5kDa_-cholane-encapsulated formulation. Porcine retinas were treated for 4 DIV with increasing concentrations of mPEG_5kDa_-cholane-encapsulated ML240, ranging from 1 µM to 150 µM (Fig. 3) to detect possible acute adverse effects under conditions where retinal structure was still well preserved. No morphological alterations or signs of cytotoxicity were observed at concentrations up to 10 µM compared to control conditions, supporting a favorable safety profile at therapeutically relevant doses. Quantification of ONL thickness revealed that only at the highest concentrations tested (50 µM and 150 µM) was there a slight decrease in the number of ONL rows compared to the 1 µM group (Fig. 3B), although this decrease was not statistically significant compared to the vehicle control.

While the control group showed substantial variability, the percentage of TUNEL-positive cells in the ONL remained consistently low across all tested concentrations up to 50 µM (Fig. 3C). A modest increase in TUNEL labeling was observed at 150 µM; however, it did not reach statistical significance, likely due to the high variability within the control group.

### mPEG_5kDa_-cholane-encapsulated ML240 is well tolerated in monkey and human retinal explants

**Fig. 4:**
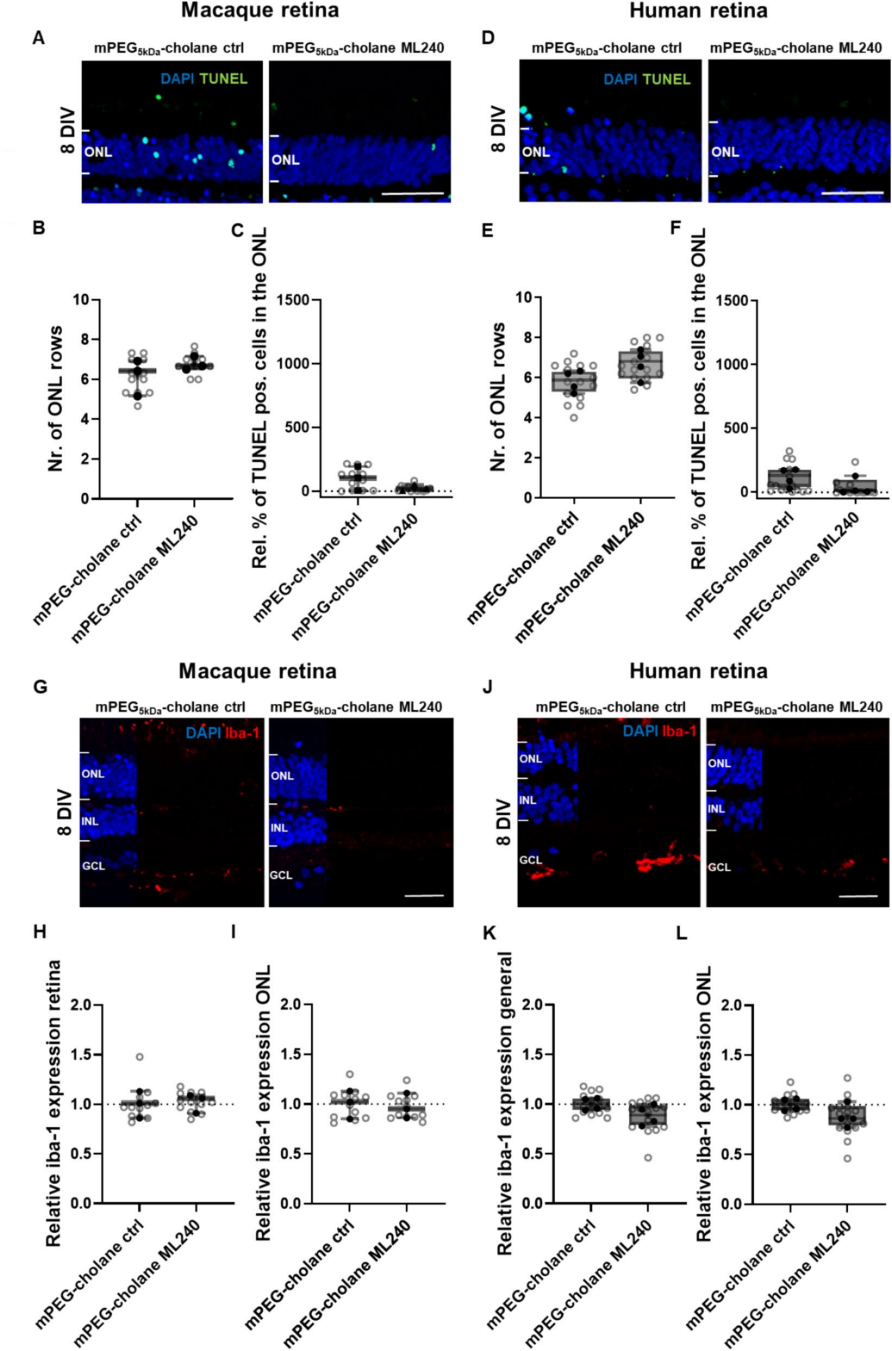
Effect of VCP inhibition in monkey and human retinal explants in vitro. Monkey and human retinal explants were cultured for 8 DIV and treated with mPEG_5kDa_-cholane-encapsulated ML240 (5 µM) or corresponding controls. Retina sections were stained using the TUNEL assay as a cell death marker and DAPI for nuclei counterstaining (**A**, **D**). Microglial activation was visualized by Iba1 immunostaining and DAPI as nuclei counterstaining (**G**, **J**). Neuroprotection was evaluated by quantifying the number of photoreceptor nuclei rows in the ONL (**B**, **E**) and the percentage of TUNEL-positive cells in the ONL (**C**, **F**). Microglial activation was assessed by measuring Iba1 fluorescence intensity across the entire retinal section (**H**, **K**) and specifically in the ONL (**I**, **L**), normalized to the respective controls. Iba1-positive microglia were predominantly localized in the GCL and IPL, consistent with their normal distribution in primate retina [36–38]. The average value of each retina explant is represented as filled circles, while measurements of different retinal sections within the explants are represented as open circles. Statistical analysis was performed using an unpaired *t*-test (*n* = 3–4 independent cultures; monkey samples were derived from explants obtained from both eyes of the same animal, human samples from one eye per donor). Scale bar: 50 µm

To extend our evaluation to the primate retina, the effects of mPEG_5kDa_-cholane-encapsulated ML240 were assessed in retinal explants from adult monkeys and human donors without known disease-causing mutations. Compared to porcine retinal explants, monkey and human explants exhibited markedly reduced degeneration under in vitro conditions, as indicated by the lower numbers of TUNEL-positive cells in the outer retina of both species (Fig. 4A, D compared to Fig. 1D). This reduced degeneration was accompanied by a milder inflammatory response, reflected by lower Iba1 expression in retinal microglia (Fig. 4G, J, compared to Fig. 2D). Due to the minimal degeneration observed in monkey and human explants under these culture conditions, and the reduced sample size, the evaluation of neuroprotective effects was inherently limited. Treatment with mPEG_5kDa_-cholane ML240 (5 µM) showed a non-significant reduction in TUNEL-positive cells in monkey explants (Fig. 4C), and no significant changes were detected in ONL thickness (Fig. 4B, E). In human retinal explants, TUNEL-positive cells were extremely scarce in both control and ML240-treated conditions (Fig. 4F). Although a slight, non-significant increase in ONL thickness was observed following treatment with mPEG_5kDa_-cholane ML240 (Fig. 4E), this difference did not reach statistical significance. Nevertheless, both models allowed for confirmation of the absence of cytotoxicity.

In porcine retinal explants, Iba1-positive microglia were distributed throughout the retina, including the outer nuclear and plexiform layers, consistent with the stronger degeneration observed in this species under culture conditions. In contrast, macaque and human explants displayed Iba1-positive cells mainly in the ganglion cell and inner plexiform layers, reflecting the characteristic localization of microglia in healthy primate and human retina [36–40] (Fig. 4H, I, K, L). Importantly, no significant increase in Iba1 expression was observed in either species across retinal layers following treatment, indicating an absence of treatment-induced inflammation. Similar results were observed when explants from both species were treated with ML240 dissolved in DMSO (Additional file 1: Fig. S5). Consistent with porcine findings, neither rhodopsin distribution nor cone density was altered by VCP inhibition in monkey or human retinal explants (Additional file 1: Figs. S6 and S7).

**Fig. 5:**
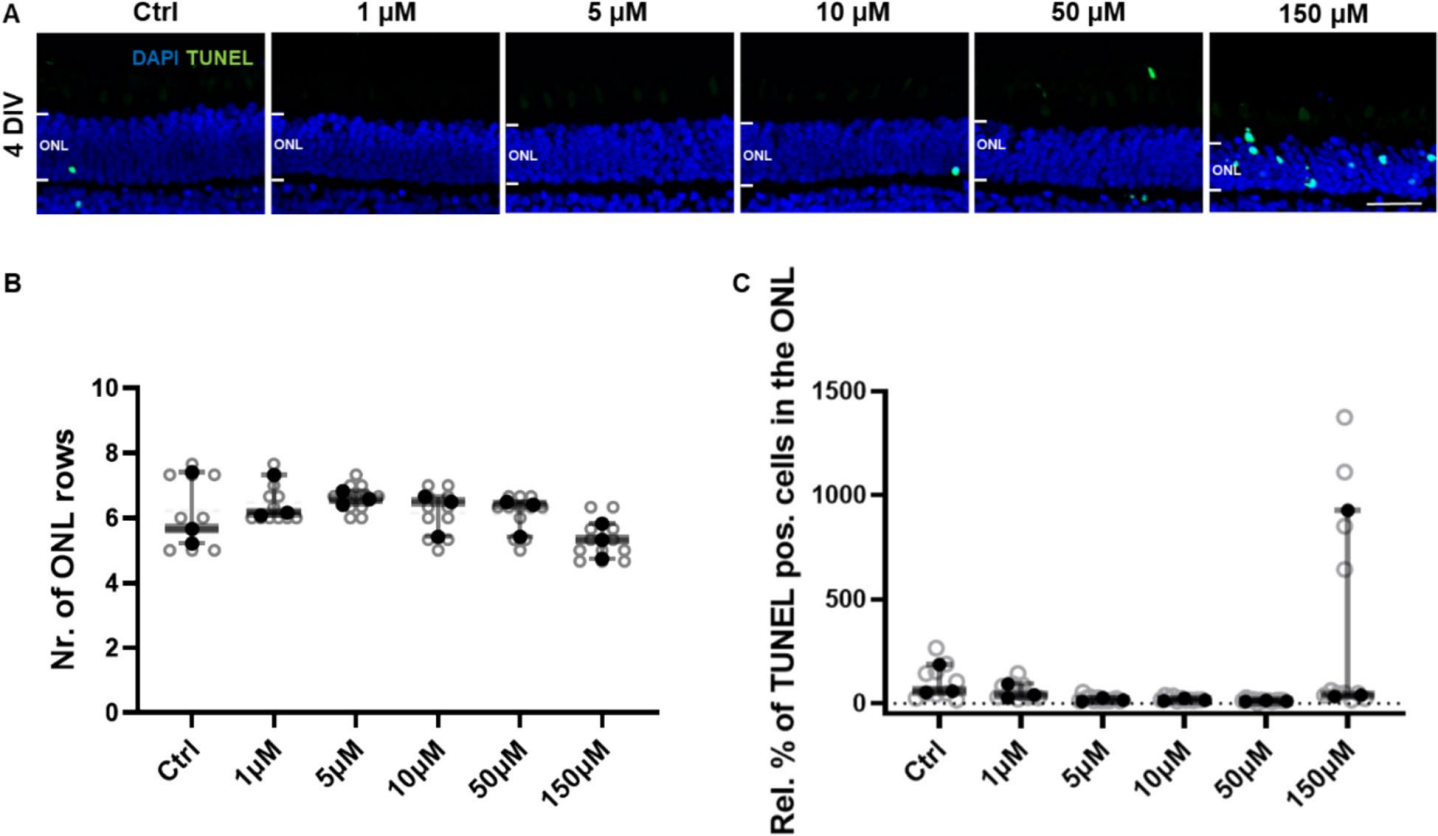
Dose escalation of mPEG_5kDa_-cholane-encapsulated ML240 in monkey retinal explants cultured for 4 DIV. **A** Monkey retinal explants were treated with increasing concentrations of mPEG_5kDa_-cholane-encapsulated ML240: 1 µM, 5 µM, 10 µM, 50 µM, 150 µM. Empty mPEG_5kDa_-cholane nanoparticles were used as controls at the same concentration as the highest dose used (7.5 mg/mL). Photoreceptor cell death was assessed by TUNEL assay, with DAPI as a nuclear counterstain. The average of each retinal explant is represented as filled circles, while measurements from different retinal sections within the same explant are represented as open circles. Statistical analysis was performed using one-way ANOVA followed by Tukey’s multiple comparisons test (*n* = 3 independent cultures; monkey samples were derived from explants obtained from both eyes of the same animal). Scale bar: 50 µm

To further explore dose tolerability, a dose-escalation experiment with mPEG5kDa-cholane-encapsulated ML240 was carried out in monkey retinal explants. The obtained results were consistent with those obtained from the porcine retina (Fig. 5). No signs of cytotoxicity were observed at concentrations up to 50 µM. At the highest dose tested (150 µM), a modest but non-significant decrease in ONL thickness was observed (Fig. 5B), accompanied by a slight increase in the percentage of TUNEL-positive cells (Fig. 5C); however, these differences did not reach statistical significance.

**Fig. 6:**
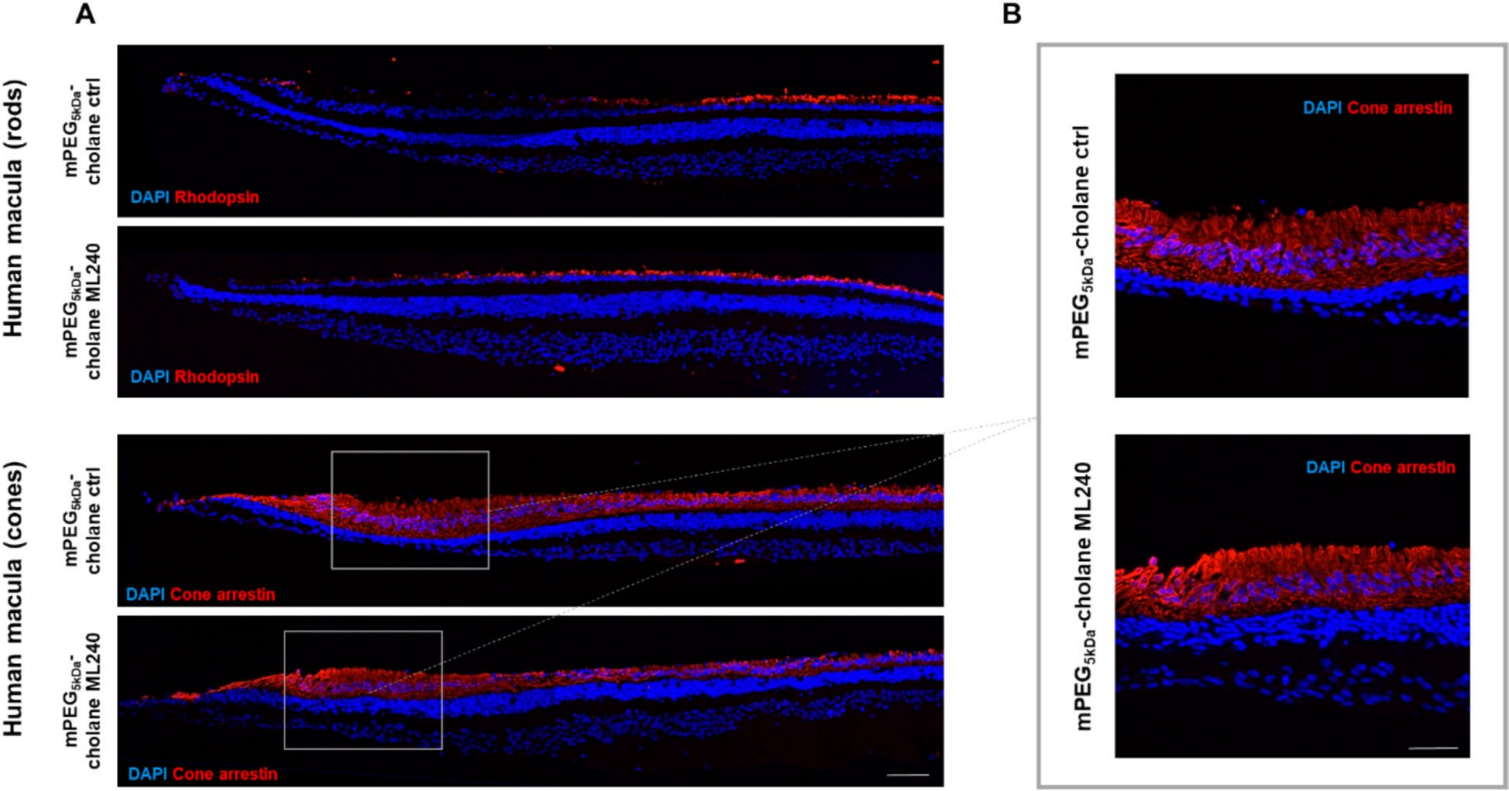
Effect of VCP inhibition on the human macula and fovea centralis. Macular regions from human retinal explants were cultured for 8 DIV and treated with mPEG_5kDa_-cholane encapsulated ML240 (5 µM) or mPEG_5kDa_-cholane empty nanoparticle control. **A** Rhodopsin immunostaining was used to visualize the distribution of rod photoreceptors, and cone arrestin immunostaining was used to visualize the cone photoreceptors. **B** Higher magnification images of the fovea centralis show the densely packed cone region characteristic of the human macula. Scale bars: 100 µm (**A**), 50 µm (**B**)

In one of the human donor eyes, we were able to successfully identify and isolate the macular region, including the fovea centralis and parafovea (Fig. 6). These sub-regions differ in photoreceptor composition, as confirmed by immunofluorescence staining for rhodopsin and cone arrestin (Fig. 6A). The fovea centralis (Fig. 6B), composed exclusively of cones, showed minimal rhodopsin and strong cone arrestin labeling, while rhodopsin expression increased progressively toward the parafovea.

Importantly, no cytotoxic effects were observed in macular sections treated with mPEG_5kDa_-cholane-encapsulated ML240 5 µM compared to controls (Fig. 6B), suggesting preserved tissue integrity in cone-rich central regions. Thus, these data demonstrate that ML240 is well tolerated in non-human primate and human retinal explants, including the macula. Although neuroprotective effects could not be substantiated under these conditions, the absence of cytotoxicity and structural alterations supports the safety profile of ML240 in higher-order retinal models.

### VCP inhibition by ML240 does not affect inner retinal neurons in monkey and human retinas in vitro

In addition to evaluating photoreceptor integrity, the cellular safety profile of ML240 was assessed in relation to other major retinal neuronal populations. This is particularly relevant for clinical translation, as damage to inner retinal cells, such as bipolar, horizontal, amacrine, and ganglion cells, could compromise overall visual processing even in the absence of photoreceptor degeneration.

**Fig. 7:**
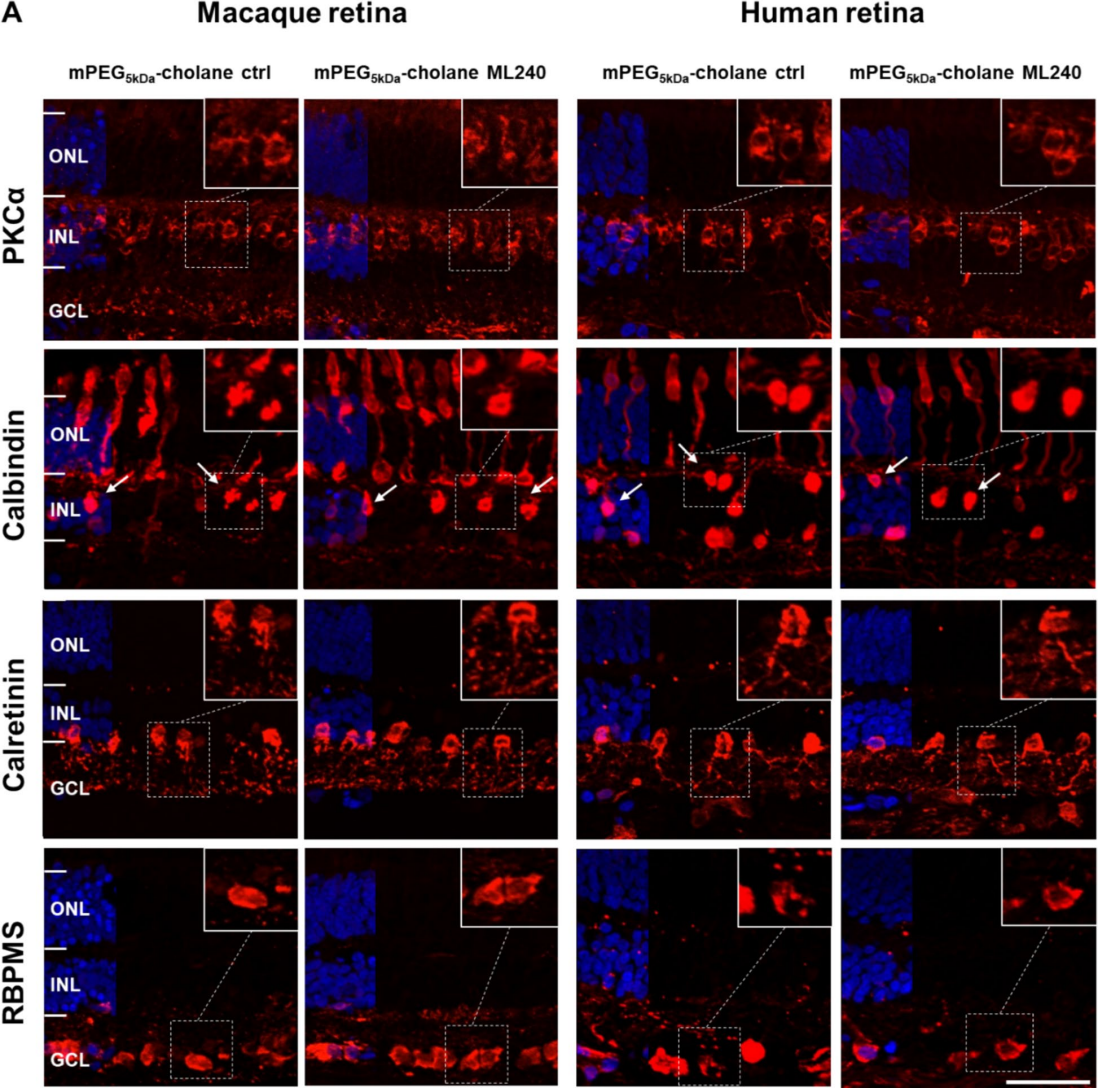
VCP inhibition by ML240 does not affect other retinal cell types in macaque and human explants in vitro. Macaque and human retinal explants were cultured and treated with mPEG_5kDa_-cholane-encapsulated ML240 (5 µM) or vehicle control for 8 DIV. **A** Inner retinal neurons were visualized by immunofluorescence staining with markers for bipolar cells (PCKα, first row), horizontal cells (calbindin, indicated by white arrows, second row), amacrine cells (calretinin, third row), and ganglion cells (RBPMS, fourth row). DAPI was used for nuclei counterstaining. Higher magnification images of the different cell types are presented in the upper right corner of every image. No differences were observed in terms of inner retinal structure and cell morphology between treated groups and vehicle control groups. Scale bar: 50 µm

We performed immunofluorescence staining for bipolar (PKCα), horizontal (calbindin), amacrine (calretinin), and ganglion cells (RBPMS) in monkey and human retinal explants cultured for 8 days (8 DIV) and treated with mPEG_5kDa_-cholane-encapsulated ML240 (5 µM) or vehicle control (Fig. 7). No morphological alterations or cytotoxic effects were observed in any of the retinal types examined following ML240 treatment. These results were consistent with findings from explants treated with ML240 dissolved in DMSO (Additional file 1: Fig. S8). Taken together, these data indicate that ML240, at the tested dose, is non-toxic to a broad range of retinal neuronal populations.

## Discussion

This study presents a comprehensive preclinical evaluation of the retinal safety and potential neuroprotective properties of the VCP inhibitor ML240 using organotypic retinal explants from porcine, non-human primate, and human donors, including central regions such as the macula and fovea. The investigation encompassed both the free form of ML240 and its encapsulation within mPEG_5kDa_-cholane nanocarriers, facilitating ex vivo analysis of dose-dependent effects and safety margins.

The porcine retina served as a model of general retinal degeneration rather than a photoreceptor-specific one, allowing quantitative assessment of structural protection in the ONL. In contrast, macaque and human explants were used primarily to assess retinal safety and tolerability. Experimental induction of degeneration in these species was avoided due to the limited availability of donor tissue and because chemically or pharmacologically induced insults do not faithfully reproduce the complexity of inherited retinal degenerations. Previous studies from our group have already demonstrated the neuroprotective efficacy of VCP inhibition in several murine models of retinal degeneration, providing the rationale for focusing here on cross-species safety evaluation.

The study demonstrates that ML240 exhibits a favorable safety profile across all species tested. No cytotoxic effects or retinal disorganization were observed at concentrations up to 50 µM. Both photoreceptors and inner retinal layers, including bipolar, horizontal, amacrine, and ganglion cells, remained structurally intact and morphologically unaffected by treatment, supporting the retina-wide tolerability of ML240 beyond photoreceptor preservation. Furthermore, ML240 did not induce inflammation, a common adverse event in retinal therapy development [41]. Conversely, expression of Iba1, a marker of microglial activation [42], was either stable or reduced in treated explants, particularly when ML240 was delivered in its encapsulated form, suggesting a potential anti-inflammatory effect. The localization of Iba1-positive microglia differed between species, paralleling the degree of retinal degeneration. In porcine explants, which undergo more pronounced culture-related stress, microglia extended into outer retinal layers, in agreement with previous observations in degenerating porcine retina [35]. Conversely, in macaque and human explants, microglia remained confined to the inner retina, matching the distribution reported for healthy primate and human retinas [36, 37, 39]. This suggests that the Iba1 staining pattern reflects physiological species-specific organization and tissue condition.

Encapsulation of ML240 in mPEG_5kDa_-cholane improved the specific activity of the compound in porcine explants, allowing a dose reduction from 20 µM (free form) to 5 µM (encapsulated), in agreement with previous findings in murine models [32]. In the current study, we expanded on this by including a dose-escalation assessment in more human-relevant models, showing that the treatment was well tolerated up to 50 µM (10 × the therapeutic dose), with only minimal adverse effects observed at 150 µM in porcine and macaque retinal explants. ML240 also demonstrated favorable tolerability in central retinal regions. No indications of cytotoxicity were observed in human macular explants, including the fovea. Furthermore, cone outer segments appeared more compact and organized in ML240-treated samples, supporting the preservation of cone-rich central retinal structure and confirming tissue integrity in these high-acuity regions.

Although organotypic retinal explants maintain significant aspects of the native retinal architecture and cell–cell interactions, they are severed from the optic nerve (axotomy) and lack blood circulation. These conditions, combined with preparation-related stress and the ex vivo environment, lead to progressive photoreceptor degeneration, even in healthy WT tissue [22, 43]. In porcine explants, degeneration was clearly apparent and of greater variability than in macaque and human tissue, where signs of photoreceptor degeneration were subtle. The fact that ML240 could slow down degeneration in a retina not affected by mutation suggests that ML240 may protect in a mutation-independent manner, making it a candidate treatment for different forms of retinal degeneration. The likely reason is that perturbed proteostasis, oxidative stress, and inflammation are features of retinal degeneration that occur regardless of the initiating mutation [44]. By acting on these common pathological pathways, ML240 may offer therapeutic benefit not only for IRDs but also for other retinal conditions such as AMD and glaucoma [45, 46].

Among the limitations of this study are the absence of a disease-driving mutation in the large animals studied and the limited availability of macaque and human retinal tissue, resulting in only small numbers of biological replicates for some experiments. These limitations, together with the minimal degeneration observed in primate and human explants, precluded definitive assessment of neuroprotective efficacy in those models, and, therefore, these results should be interpreted as exploratory. Nevertheless, the consistency of the findings across three species, including non-human primates and human donors, strengthens the findings related here.

Future studies should confirm the therapeutic potential of ML240 in vivo under physiological conditions. Optimized delivery strategies, such as intravitreal injections or sustained-release systems, will be necessary to ensure sustained and targeted drug exposure. Evaluation of pharmacokinetics, bioavailability, and regulatory requirements will be pivotal steps toward clinical translation [47, 48]. In addition, future studies may determine whether ML240 may be a suitable candidate for combination therapy, potentially enhancing the efficacy of gene therapies or anti-inflammatory agents.

## Conclusions

In summary, ML240 exhibits a strong safety profile in the primate retina, including the macula, and the results of this study support its further development as a mutation-independent therapeutic candidate for genetically diverse forms of retinal degeneration.

## Supplementary Information

Additional file 1: Fig. S1. Representative images of full-thickness porcine retinal sections after culture for 8 days with the different treatment conditions. Fig. S2. Rhodopsin distribution in porcine retinal explants is not altered by VCP inhibition. Fig. S3. VCP inhibition does not affect the survival of porcine cone photoreceptors. Fig. S4. Iba1 protein expression in porcine retinal explants. Fig. S5. Effect of VCP inhibition by free ML240 in macaque and human retinal explants*.* Fig. S6. Rod outer segments and rhodopsin localization are not altered by VCP inhibition in macaque and human retinal explants. Fig. S7. VCP inhibition does not affect macaque or human cone photoreceptor survival. Fig. S8. VCP inhibition by free ML240 does not affect other retinal cell types in macaque and human explants in vitro.

Additional file 2. Original uncropped gels of western blot to detect the expression of Iba1 and β-actin as loading control in porcine retinal explants homogenates.

## Data Availability

The datasets used and/or analysed during the current study are available from the corresponding author on reasonable request.

## Abbreviations

AMD: Age-related macular degeneration
CNS: Central nervous system
DIV: Days in vitro
DMSO: Dimethyl sulfoxide
FAZ: Foveal avascular zone
FITC: Fluorescein isothiocyanate
GCL: Ganglion cell layer
IRDs: Inherited retinal diseases
mPEG_5kDa_-cholane: Methoxy polyethylene glycol 5 kDa-cholane
NHPs: Non-human primates
ONL: Outer nuclear layer
OS: Outer segment
PB: Phosphate buffer
PBS: Phosphate-buffered saline
PR: Photoreceptor
RP: Retinitis pigmentosa
VCP: Valosin-containing protein

## References

[1] Ptito M, Bleau M, Bouskila J. The retina: a window into the brain. Cells. 2021. 10.3390/cells10123269. 34943777 10.3390/cells10123269 PMC8699497

[2] Masland RH. The fundamental plan of the retina. Nat Neurosci. 2001;4(9):877–86. 11528418 10.1038/nn0901-877

[3] Pardue MT, Allen RS. Neuroprotective strategies for retinal disease. Prog Retin Eye Res. 2018;65:50–76. 29481975 10.1016/j.preteyeres.2018.02.002 PMC6081194

[4] Georgiou M, Fujinami K, Michaelides M. Inherited retinal diseases: therapeutics, clinical trials and end points—a review. Clin Exp Ophthalmol. 2021;49(3):270–88. 33686777 10.1111/ceo.13917

[5] Wong CH, Siah KW, Lo AW. Estimation of clinical trial success rates and related parameters. Biostatistics. 2019;20(2):273–86. 29394327 10.1093/biostatistics/kxx069 PMC6409418

[6] Kim E, Yang J, Park S, Shin K. Factors affecting success of new drug clinical trials. Ther Innov Regul Sci. 2023;57(4):737–50. 37166743 10.1007/s43441-023-00509-1 PMC10173933

[7] Steeves JD. Bench to bedside: challenges of clinical translation. Prog Brain Res. 2015;218:227–39. 25890140 10.1016/bs.pbr.2014.12.008

[8] Baden T, Euler T, Berens P. Understanding the retinal basis of vision across species. Nat Rev Neurosci. 2020;21(1):5–20. 31780820 10.1038/s41583-019-0242-1

[9] Fletcher EL, Jobling AI, Vessey KA, Luu C, Guymer RH, Baird PN. Animal models of retinal disease. Prog Mol Biol Transl Sci. 2011;100:211–86. 21377628 10.1016/B978-0-12-384878-9.00006-6

[10] Haverkamp S, Wässle H. Immunocytochemical analysis of the mouse retina. J Comp Neurol. 2000;424(1):1–23. 10888735

[11] Ortín-Martínez A, Nadal-Nicolás FM, Jiménez-López M, Alburquerque-Béjar JJ, Nieto-López L, García-Ayuso D, et al. Number and distribution of mouse retinal cone photoreceptors: differences between an albino (Swiss) and a pigmented (C57/BL6) strain. PLoS One. 2014;9(7):e102392. 25029531 10.1371/journal.pone.0102392 PMC4100816

[12] Jakobsen TS, Fabian-Jessing BK, Hansen S, Bek T, Askou AL, Corydon TJ. Porcine models of choroidal neovascularization: a systematic review. Exp Eye Res. 2023. 10.1016/j.exer.2023.109590. 37474015 10.1016/j.exer.2023.109590

[13] Chandler, Samuelson, Mackay. Photoreceptor density of the domestic pig retina. Vet Ophthalmol. 1999;2(3):179–84. 10.1046/j.1463-5224.1999.00077.x 11397262

[14] Dufour VL, Aguirre GD. Canine models of inherited retinal diseases: from neglect to well-recognized translational value. Mamm Genome. 2024. 10.1007/s00335-024-10091-y. 39739008 10.1007/s00335-024-10091-y PMC12129671

[15] Seah I, Goh D, Chan HW, Su X. Developing non-human primate models of inherited retinal diseases. Genes. 2022;13(2):344. 35205388 10.3390/genes13020344 PMC8872446

[16] Ferla R, Pugni E, Lupo M, Tiberi P, Fioretto F, Perota A, et al. Retinal gene therapy for Stargardt disease with dual AAV intein vectors is both safe and effective in large animal models. Sci Adv. 2025;11(13):eadt9354. 40138422 10.1126/sciadv.adt9354 PMC11939046

[17] Marshall LJ, Bailey J, Cassotta M, Herrmann K, Pistollato F. Poor translatability of biomedical research using animals—a narrative review. Altern Lab Anim. 2023;51(2):102–35. 36883244 10.1177/02611929231157756

[18] McGonigle P, Ruggeri B. Animal models of human disease: challenges in enabling translation. Biochem Pharmacol. 2014;87(1):162–71. 23954708 10.1016/j.bcp.2013.08.006

[19] Schnichels S, Kiebler T, Hurst J, Maliha AM, Löscher M, Dick HB, et al. Retinal organ cultures as alternative research models. Altern Lab Anim. 2019;47(1):19–29. 31237165 10.1177/0261192919840092

[20] Xu W, Dong Y, Li Y, Hu Z, Paquet-Durand F, Jiao K. Organotypic retinal explant cultures from Macaque monkey. J Vis Exp. 2022;186:e64178. 10.3791/64178 36094256

[21] Carter D, Dick A. Lipopolysaccharide/interferon-γ and not transforming growth factor β inhibits retinal microglial migration from retinal explant. Br J Ophthalmol. 2003;87(4):481–7. 12642315 10.1136/bjo.87.4.481 PMC1771595

[22] Niyadurupola N, Sidaway P, Osborne A, Broadway DC, Sanderson J. The development of human organotypic retinal cultures (HORCs) to study retinal neurodegeneration. Br J Ophthalmol. 2011;95(5):720–6. 21169273 10.1136/bjo.2010.181404

[23] Muller A, Sullivan J, Schwarzer W, Wang M, Park-Windhol C, Hasler PW, et al. High-efficiency base editing in the retina in primates and human tissues. Nat Med. 2025;31(2):490–501. 39779923 10.1038/s41591-024-03422-8 PMC11835749

[24] Hasegawa T, Muraoka Y, Ikeda HO, Tsuruyama T, Kondo M, Terasaki H, et al. Neuoroprotective efficacies by KUS121, a VCP modulator, on animal models of retinal degeneration. Sci Rep. 2016;6(1):31184. 27503804 10.1038/srep31184 PMC4977562

[25] Arango-Gonzalez B, Sen M, Guarascio R, Ziaka K, del Amo EM, Hau K, et al. Inhibition of VCP preserves retinal structure and function in autosomal dominant retinal degeneration. BioRxiv. 2020:2020.11. 17.384669.

[26] Sen M, Kutsyr O, Cao B, Bolz S, Arango-Gonzalez B, Ueffing M. Pharmacological inhibition of the VCP/proteasome axis rescues photoreceptor degeneration in RHOP23H rat retinal explants. Biomolecules. 2021;11(10):1528. 34680161 10.3390/biom11101528 PMC8534135

[27] Yeo BK, Yu S-W. Valosin-containing protein (VCP): structure, functions, and implications in neurodegenerative diseases. Anim Cells Syst. 2016;20(6):303–9.

[28] Braun RJ, Zischka H. Mechanisms of Cdc48/VCP-mediated cell death—from yeast apoptosis to human disease. Biochimica et Biophysica Acta (BBA). 2008;1783(7):1418–35. 18284922 10.1016/j.bbamcr.2008.01.015

[29] Griciuc A, Aron L, Roux MJ, Klein R, Giangrande A, Ueffing M. Inactivation of VCP/ter94 suppresses retinal pathology caused by misfolded rhodopsin in *Drosophila*. PLoS Genet. 2010;6(8):e1001075. 20865169 10.1371/journal.pgen.1001075 PMC2928793

[30] Chou TF, Li K, Frankowski KJ, Schoenen FJ, Deshaies RJ. Structure–activity relationship study reveals ML240 and ML241 as potent and selective inhibitors of p97 ATPase. ChemMedChem. 2013;8(2):297–312. 23316025 10.1002/cmdc.201200520 PMC3662613

[31] Salmaso S, Bersani S, Mastrotto F, Tonon G, Schrepfer R, Genovese S, et al. Self‐assembling nanocomposites for protein delivery: supramolecular interactions between PEG‐cholane and rh‐G‐CSF. J Control Release. 2012;162(1):176–84. 22727711 10.1016/j.jconrel.2012.06.018

[32] Sen M, Al-Amin M, Kicková E, Sadeghi A, Puranen J, Urtti A, et al. Retinal neuroprotection by controlled release of a VCP inhibitor from self-assembled nanoparticles. J Control Release. 2021;339:307–20. 34606936 10.1016/j.jconrel.2021.09.039

[33] Noel PR, Barnett KC, Davies RE, Jolly DW, Leahy JS, Mawdesley-Thomas LE, et al. The toxicity of dimethyl sulphoxide (DMSO) for the dog, pig, rat and rabbit. Toxicology. 1975;3(2):143–69. 1124535 10.1016/0300-483x(75)90081-5

[34] Blank T, Goldmann T, Koch M, Amann L, Schon C, Bonin M, et al. Early Microglia Activation Precedes Photoreceptor Degeneration in a Mouse Model of CNGB1-Linked Retinitis Pigmentosa. Front Immunol. 2017;8:1930. 29354133 10.3389/fimmu.2017.01930 PMC5760536

[35] Johansson K, Mohlin C. Microglia in cultured Porcine retina: qualitative immunohistochemical analyses of reactive microglia in the outer retina. Int J Mol Sci. 2023;24(1):871. 36614320 10.3390/ijms24010871 PMC9820911

[36] Fan W, Huang W, Chen J, Li N, Mao L, Hou S. Retinal microglia: functions and diseases. Immunology. 2022;166(3):268–86. 35403700 10.1111/imm.13479

[37] Murenu E, Gerhardt M-J, Biel M, Michalakis S. More than meets the eye: the role of microglia in healthy and diseased retina. Front Immunol. 2022;13:1006897. 36524119 10.3389/fimmu.2022.1006897 PMC9745050

[38] Reichenbach A, Bringmann A. Retinal glia. Biota Publishing; 2015.

[39] Rathnasamy G, Foulds WS, Ling E-A, Kaur C. Retinal microglia–a key player in healthy and diseased retina. Prog Neurobiol. 2019;173:18–40. 29864456 10.1016/j.pneurobio.2018.05.006

[40] Dixon MA, Greferath U, Fletcher EL, Jobling AI. The contribution of microglia to the development and maturation of the visual system. Front Cell Neurosci. 2021;15:659843. 33967697 10.3389/fncel.2021.659843 PMC8102829

[41] Mehta N, Robbins DA, Yiu G. Ocular inflammation and treatment emergent adverse events in retinal gene therapy. Int Ophthalmol Clin. 2021;61(3):151–77. 34196322 10.1097/IIO.0000000000000366 PMC8259781

[42] Imai Y, Kohsaka S. Intracellular signaling in M‐CSF‐induced microglia activation: role of Iba1. Glia. 2002;40(2):164–74. 12379904 10.1002/glia.10149

[43] Fernandez-Bueno I, Fernández-Sánchez L, Gayoso MJ, García-Gutierrez MT, Pastor JC, Cuenca N. Time course modifications in organotypic culture of human neuroretina. Exp Eye Res. 2012;104:26–38. 23022403 10.1016/j.exer.2012.08.012

[44] Bighinati A, Adani E, Stanzani A, D’Alessandro S, Marigo V. Molecular mechanisms underlying inherited photoreceptor degeneration as targets for therapeutic intervention. Front Cell Neurosci. 2024;18:1343544. 38370034 10.3389/fncel.2024.1343544 PMC10869517

[45] Roth F, Bindewald A, Holz FG. Keypathophysiologic pathways in age-related macular disease. Graefes Arch Clin Exp Ophthalmol. 2004;242:710–6. 15309554 10.1007/s00417-004-0976-x

[46] Levkovitch-Verbin H. Retinal ganglion cell apoptotic pathway in glaucoma: initiating and downstream mechanisms. Prog Brain Res. 2015;220:37–57. 26497784 10.1016/bs.pbr.2015.05.005

[47] Zhou Q, Gallo JM. The pharmacokinetic/pharmacodynamic pipeline: translating anticancer drug pharmacology to the clinic. AAPS J. 2011;13:111–20. 21246315 10.1208/s12248-011-9253-1 PMC3032092

[48] Chen M-L, Shah V, Patnaik R, Adams W, Hussain A, Conner D, et al. Bioavailability and bioequivalence: an FDA regulatory overview. Pharm Res. 2001;18:1645–50. 11785681 10.1023/a:1013319408893

