# Supplementary material for: Safety and neuroprotective efficacy of the VCP inhibitor ML240 in large-animal and human retinal explants: a preclinical ex vivo study
Source: BMC Med. 2026 Jan 10;24:54. doi: 10.1186/s12916-025-04610-0 (PMC12849125; doi:10.1186/s12916-025-04610-0)
Supplement: Supplementary file 1 — Additional file 1: Fig. S1. Representative images of full-thickness porcine retinal sections after culture for 8 days with the different treatment conditions. Fig. S2. Rhodopsin distribution in porcine retinal explants is not altered by VCP inhibition. Fig. S3. VCP inhibition does not affect the survival of porcine cone photoreceptors. Fig. S4. Iba1 protein expression in porcine retinal explants. Fig. S5. Effect of VCP inhibition by free ML240 in macaque and human retinal explants. Fig. S6. Rod outer segments and rhodopsin localization are not altered by VCP inhibition in macaque and human retinal explants. Fig. S7. VCP inhibition does not affect macaque or human cone photoreceptor survival. Fig. S8. VCP inhibition by free ML240 does not affect other retinal cell types in macaque and human explants in vitro. [file 12916_2025_4610_MOESM1_ESM.docx]

**Supplementary**

**S1. Degeneration across retinal layers in porcine explants after 8 DIV**


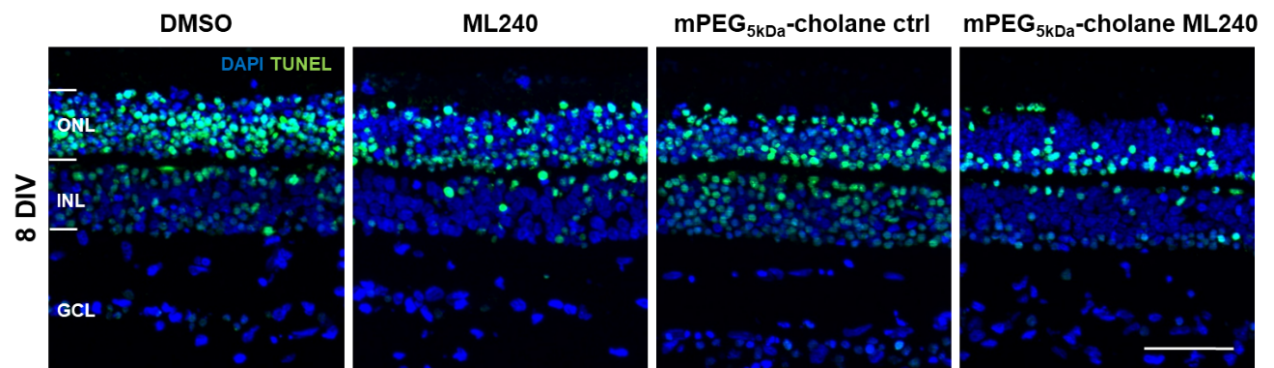


**Figure S1. Representative images of full-thickness porcine retinal sections.** Porcine retinal explants were cultured for 8 DIV and treated either with ML240 (20 µM) vs. DMSO control or with mPEG_5kDa_-cholane-encapsulated ML240 (5 µM) vs. mPEG_5kDa_-cholane control (0.25 mg/mL). TUNEL assay was used to detect photoreceptors undergoing cell death, with DAPI counterstaining for nuclear visualization. After 8 days in culture, porcine retinal explants exhibited increased cell death in all retinal layers, and treatment with VCP inhibition in its free form, as well as encapsulated in mPEG5kDa-cholane, reduced cell degeneration in all retinal layers. Scale bar: 50 µm.

**S2. ML240 does not alter rhodopsin localization in porcine explants**

**
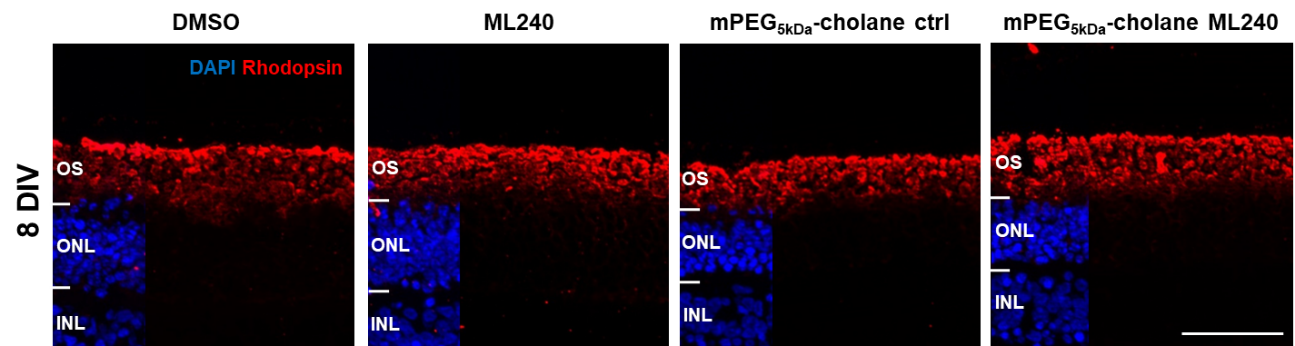
**

**Figure S2. Rhodopsin distribution does not change after VCP inhibition.** Porcine retinal explants were cultured for 8 DIV and treated either with ML240 (20 µM) *vs*. DMSO control or with mPEG_5kDa_-cholane-encapsulated ML240 (5 µM) *vs*. mPEG_5kDa_-cholane control (0.25 mg/mL). Rhodopsin immunostaining was used to visualize rod outer segments and rhodopsin localization, and DAPI was used for nuclei counterstaining. Scale bar: 50 µm.

**S3. ML240 does not affect cone photoreceptor survival in porcine explants**

**
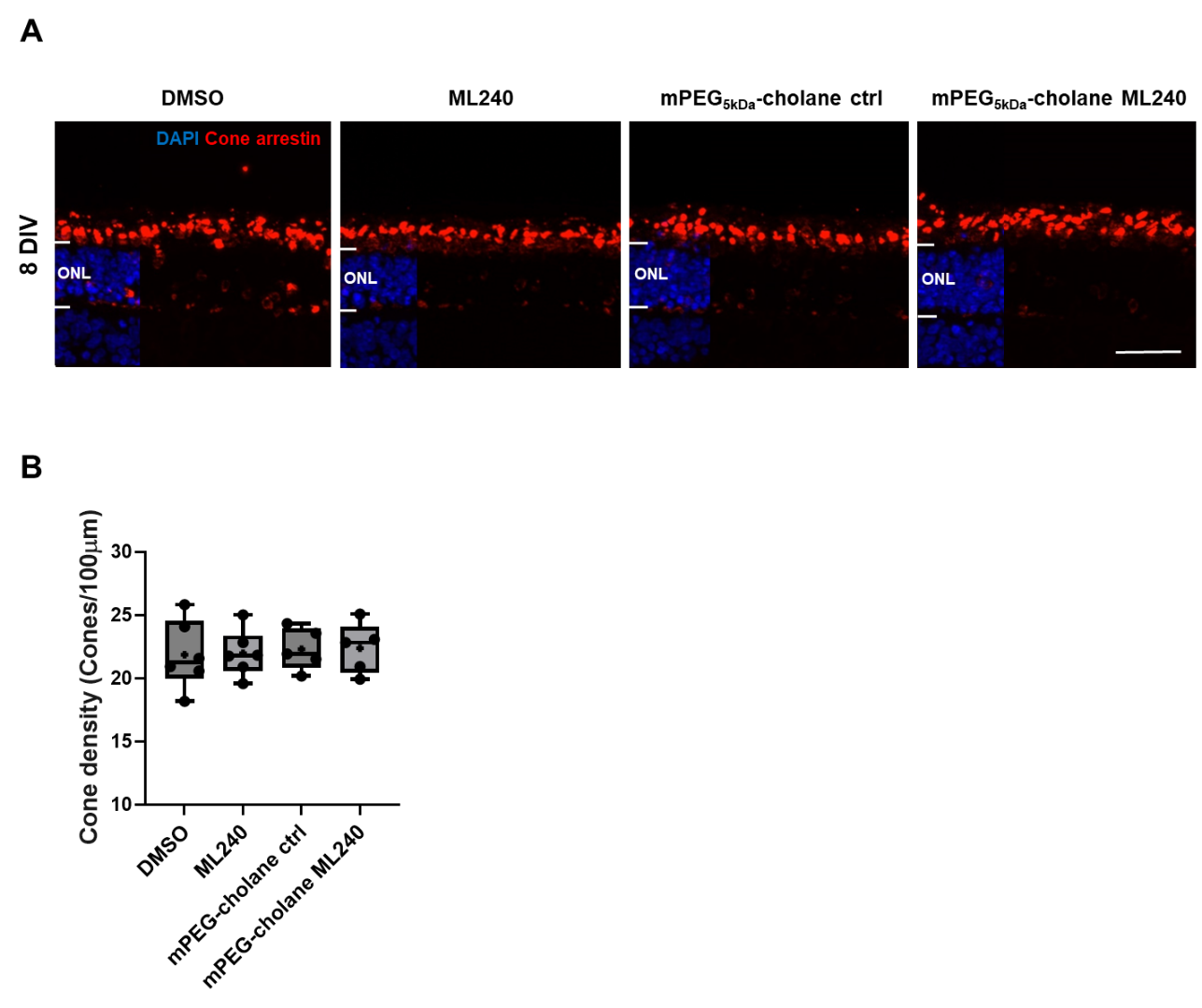
**

**Figure S3. VCP inhibition does not affect the survival of porcine cone photoreceptors.** Porcine retinal explants were cultured for 8 DIV and treated either with ML240 (20 µM) *vs*. DMSO control or with mPEG_5kDa_-cholane-encapsulated ML240 (5 µM) *vs*. mPEG_5kDa_-cholane control (0.25 mg/mL). (**A**) M opsin immunostaining was used to label cone photoreceptors, and DAPI was used for nuclei counterstaining. (**B**) Cone density was calculated by quantifying the number of cone photoreceptors per 100 µm section. Statistical analysis was performed using one-way ANOVA (n = 5-6 biological replicates). Scale bar: 50 µm.

**S4. Encapsulated ML240 reduces Iba1 protein levels in porcine explants.**


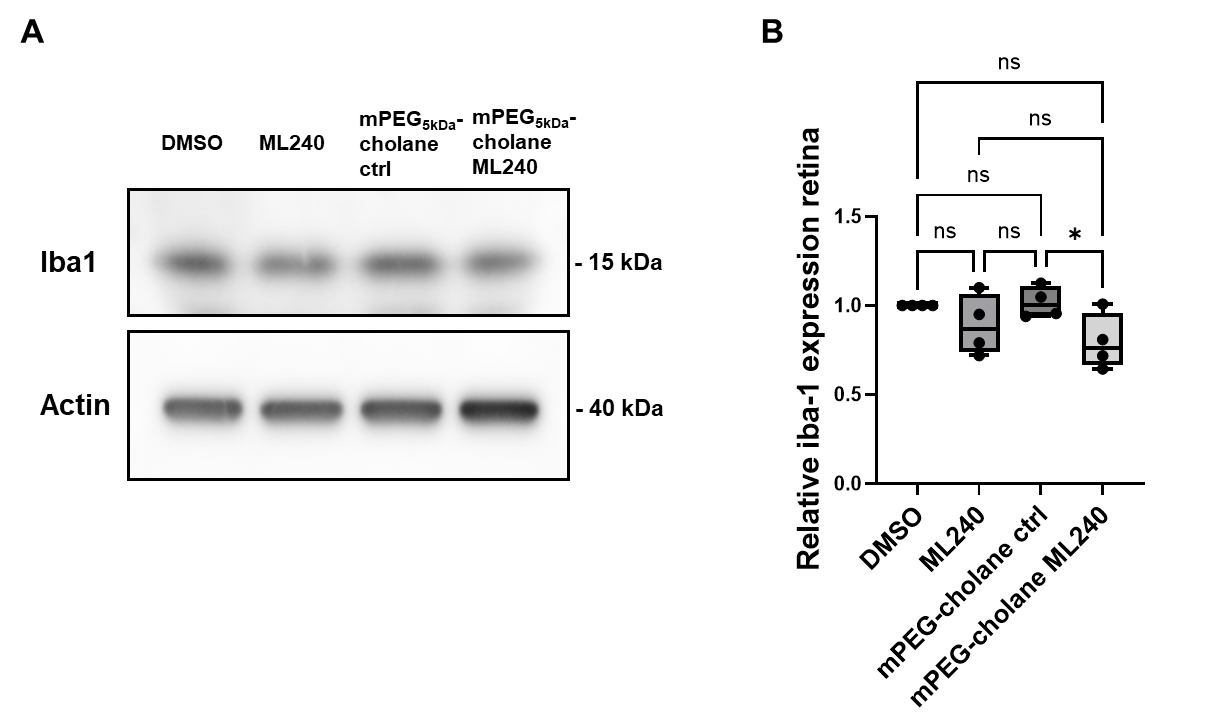


**Figure S4**. **Iba1 protein expression in porcine retinal explants.** (**A**) Representative images of western blot analysis of Iba1 protein and (**B**) relative protein expression quantification. β-actin was used as housekeeping gene for loading control. Porcine retinal explants were cultured for 8 DIV and treated with either ML240 (20 µM) or DMSO control, and mPEG_5kDa_-cholane-encapsulated ML240 (5 µM) or mPEG_5kDa_-cholane vehicle control (0.25 mg/mL). Explants treated with encapsulated ML240 showed a significant reduction of Iba1 expression with respect to the encapsulated control. Differences between treatment groups were assessed by One-way ANOVA with Tukey´s multiple comparison. * p ˂ 0.05.

**S5. ML240 shows no cytotoxicity in macaque and human retinal explants**


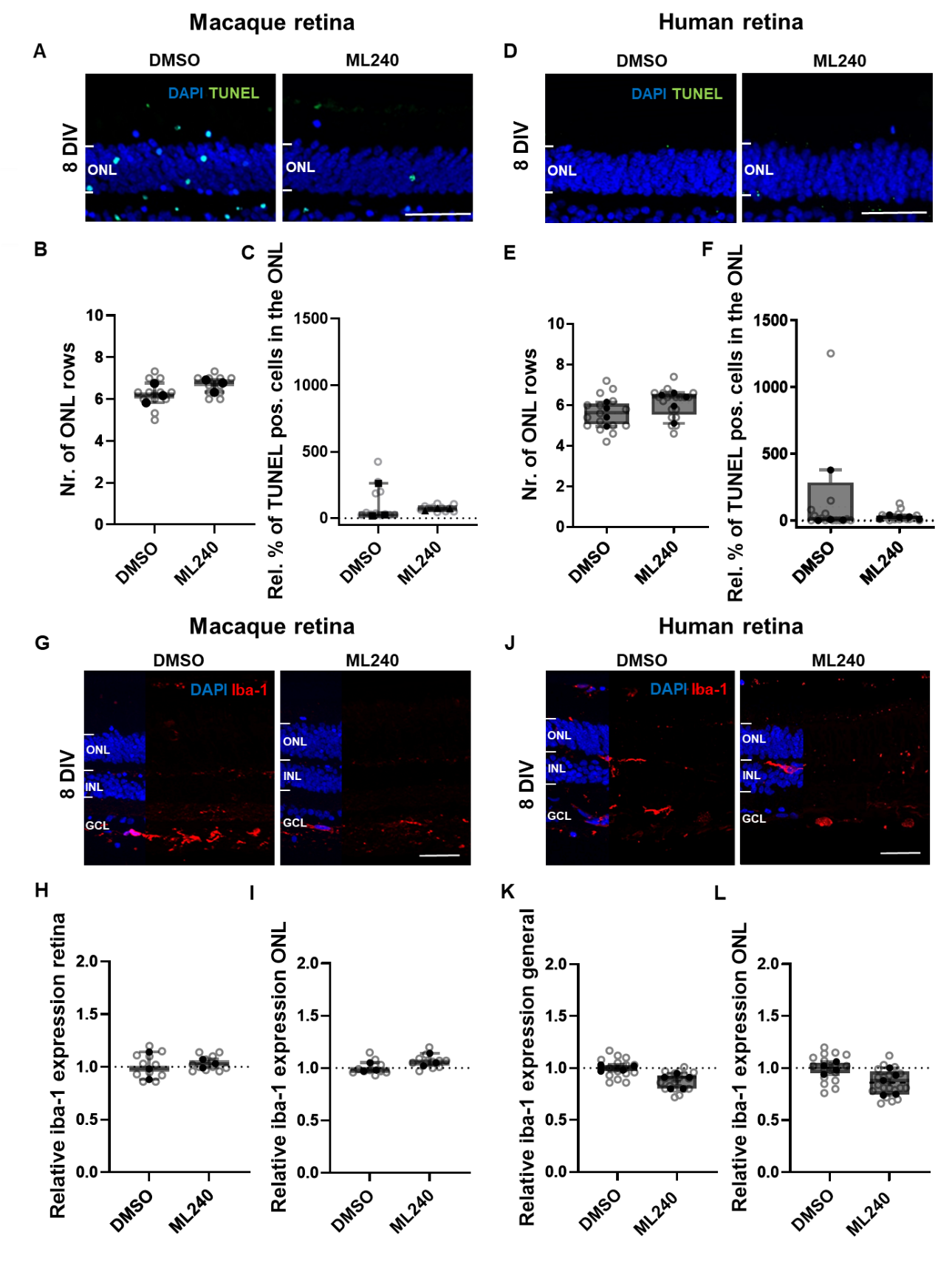


**Figure S5. Effect of VCP inhibition in macaque and human retinal explants *in vitro*.** Macaque and human retinal explants were cultured for 8 DIV and treated with ML240 (20 µM) or DMSO controls. Retina sections were stained using the TUNEL assay as a cell death marker and DAPI for nuclei counterstaining (**A, D**). Microglial activation was visualized by Iba1 immunostaining (**G, J**), and DAPI was used for nuclei counterstaining. Neuroprotection was evaluated by quantifying the number of photoreceptor nuclei rows in the ONL (**B, E**) and the percentage of TUNEL-positive cells in the ONL (**C, F**). Microglial activation was assessed by measuring Iba1 fluorescence intensity across the entire retinal section (**H, K**) and specifically in the ONL (**I, L**), normalized to the respective controls. Filled circles represent biological replicates (n = 3-5 independent cultures; monkey samples were derived from explants obtained from both eyes of the same animal, human samples from one eye per donor). Open circles represent technical replicates used to calculate means for each biological replicate. Statistical analysis was performed using an unpaired *t*-test (*p* < 0.05). Scale bar: 50 µm.

**S6. Rhodopsin distribution remains normal in macaque and human explants after ML240 treatment.**

**
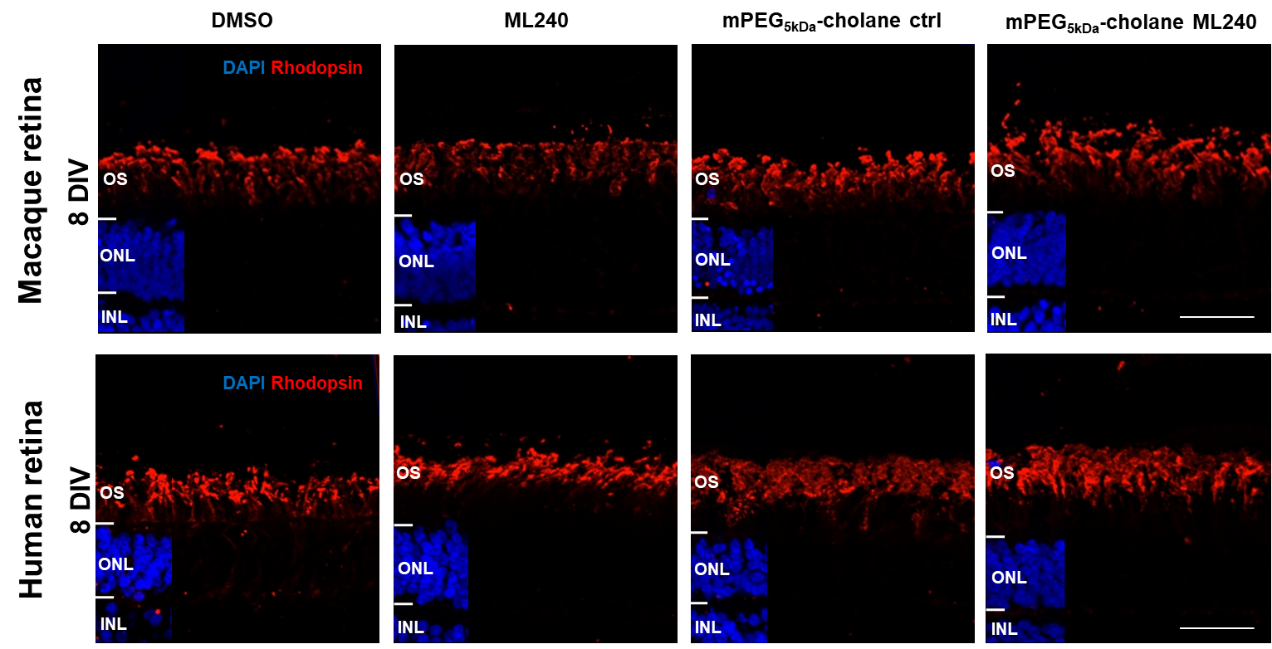
**

**Figure S6. Rod outer segments and rhodopsin localization are not altered by VCP inhibition.** Macaque and human retinal explants were cultured for 8 DIV and treated with ML240 (20 µM) *vs*. DMSO control or with mPEG_5kDa_-cholane-encapsulated ML240 (5 µM) *vs*. mPEG_5kDa_-cholane control. Rhodopsin immunostaining was used to assess rod outer segments morphology and rhodopsin distribution. DAPI was used as counterstaining. Scale bar: 50 µm.

**S7. Cone photoreceptors are preserved after ML240 treatment in macaque and human explants.**

**
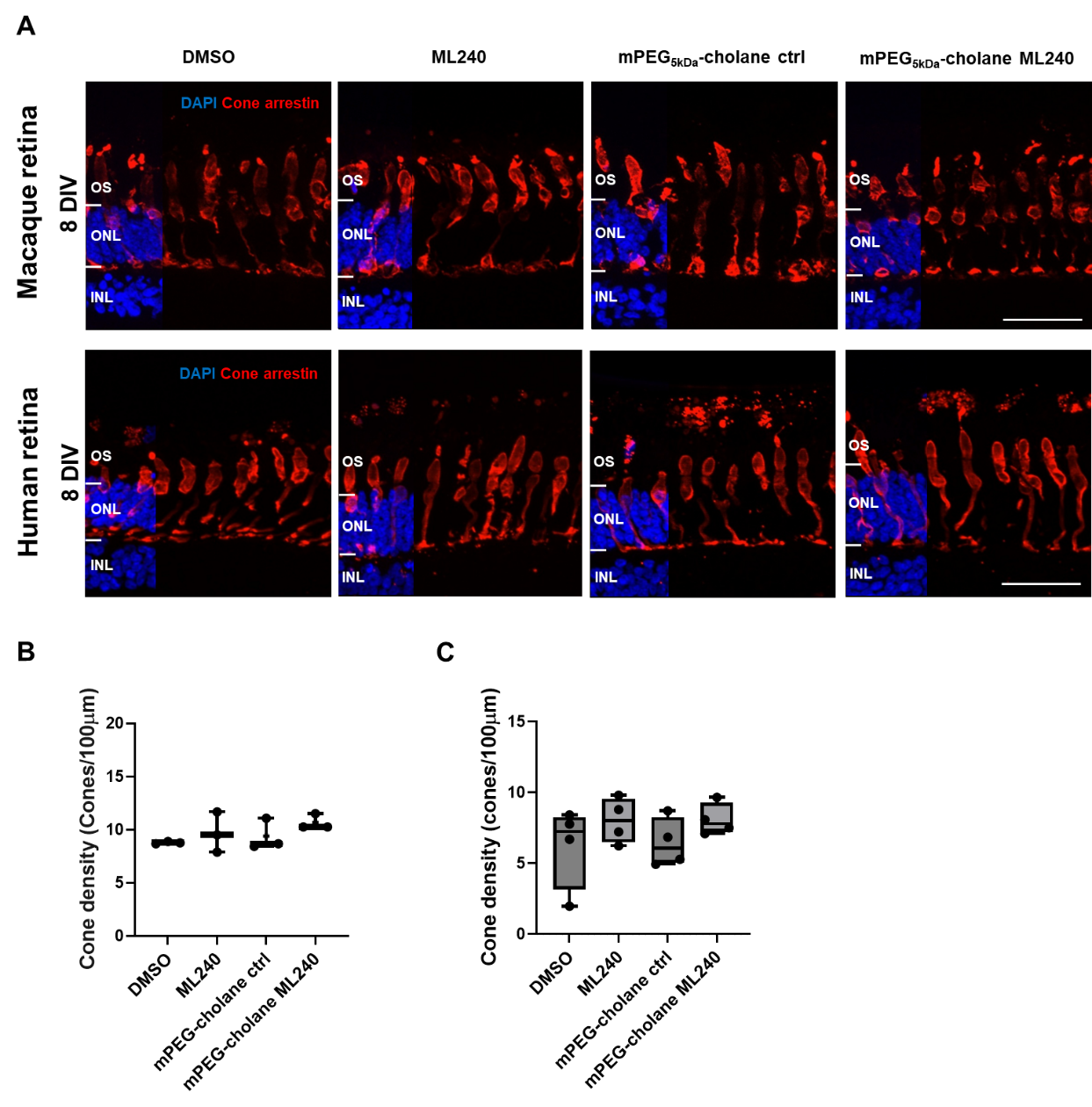
**

**Figure S7. VCP inhibition does not affect macaque or human cone photoreceptor surviva**l. Retinal explants from both species were cultured for 8 DIV and treated with ML240 (20 µM) *vs*. DMSO control or with mPEG_5kDa_-cholane-encapsulated ML240 (5 µM) *vs*. mPEG_5kDa_-cholane control. Cone arrestin immunostaining (**A**) was used to label cone photoreceptors, and DAPI was used as counterstaining. Cone density was calculated by quantifying the number of cone photoreceptors per 100 µm section in macaque explants (**B**) and human explants (**C**). One-way ANOVA was used to assess statistical significance (n = 5-6).

**S8. ML240 does not affect inner retinal cell types in macaque and human explants.**

**
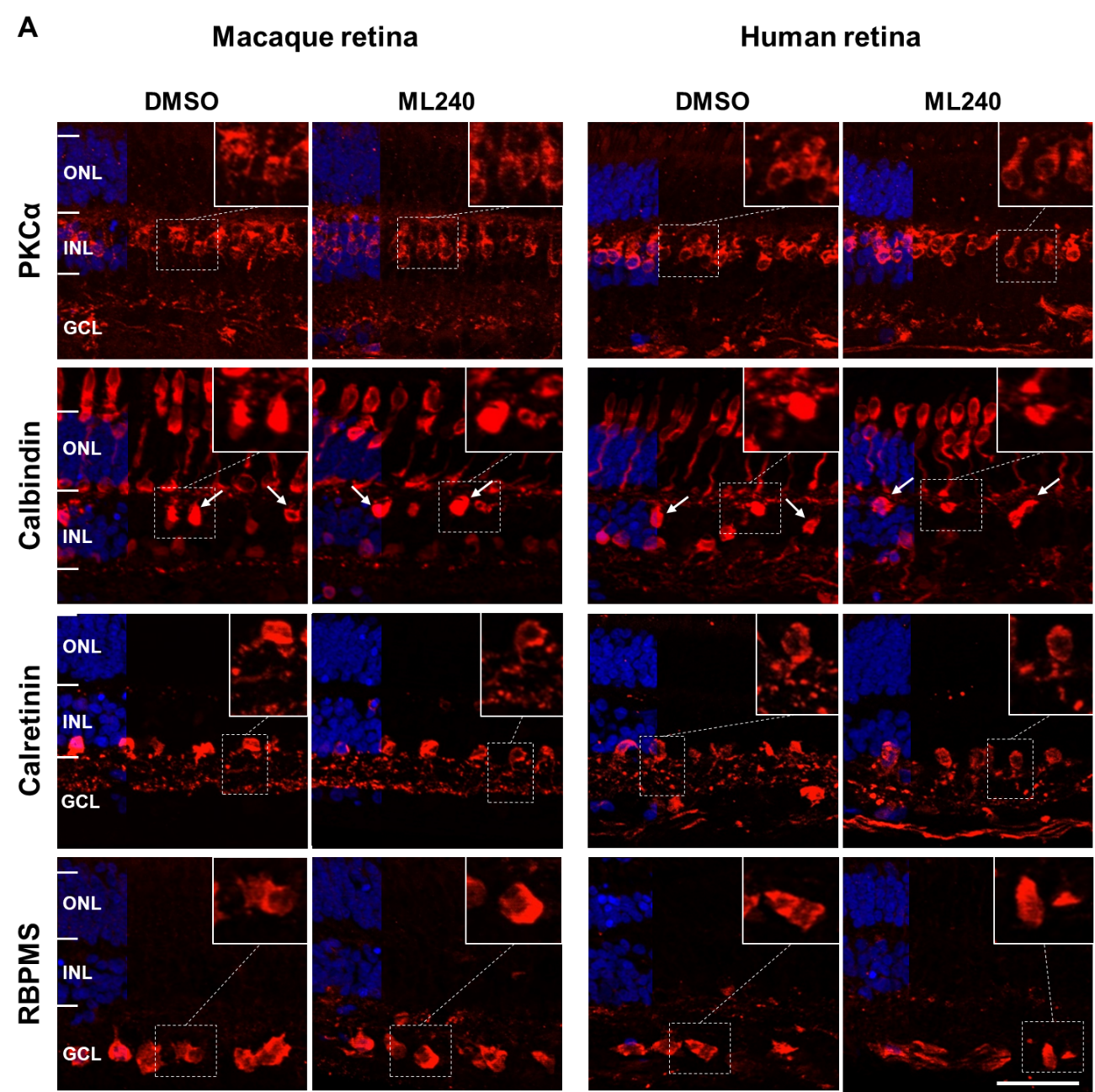
**

**Figure S8. VCP inhibition by ML240 does not affect other retinal cell types in macaque and human explants *in vitro*.** Macaque and human retinal explants were cultured and treated with ML240 (20 µM) or DMSO control for 8 DIV. (**A**) Inner retinal cells were visualized by immunofluorescence staining using specific markers for bipolar cells (PCKα, first row), horizontal cells (calbindin, second row) marked with white arrows in the images, amacrine cells (calretinin, third row), and ganglion cells (RBPMS, fourth row). DAPI was used as counterstaining. Higher-magnification insets were added to the upper right corner of each panel in the revised version to facilitate assessment of morphology. No differences were observed between treated and control explants. Scale bar: 50 µm.
