# Supplementary figures and images for: Safety and neuroprotective efficacy of the VCP inhibitor ML240 in large-animal and human retinal explants: a preclinical ex vivo study
Source: BMC Med. 2026 Jan 10;24:54. doi: 10.1186/s12916-025-04610-0 (PMC12849125; doi:10.1186/s12916-025-04610-0)

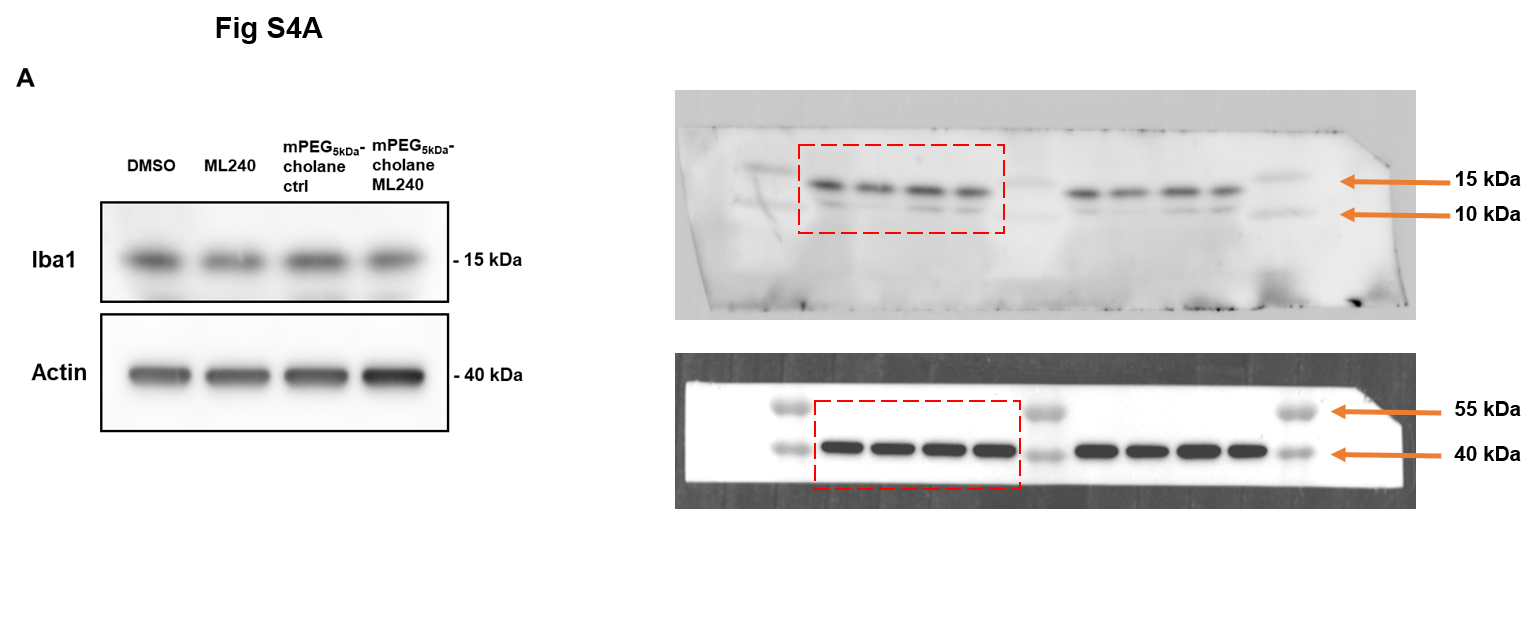

Supplement: Supplementary file 2 — Additional file 2. Original uncropped gels of western blot to detect the expression of Iba1 and β-actin as loading control in porcine retinal explants homogenates. [file 12916_2025_4610_MOESM2_ESM.png]
